# Synthesis, Antibacterial and Pharmacokinetic Evaluation of Novel Derivatives of Harmine N^9^-Cinnamic Acid

**DOI:** 10.3390/molecules26164842

**Published:** 2021-08-10

**Authors:** Yan Liang, Dian He, Deshun Zhou, Junshuai Li, Lei Tang, Zhen Wang

**Affiliations:** 1 School of Pharmacy, Lanzhou University, West Donggang Road No. 199, Lanzhou 730000, China; liangy18@lzu.edu.cn (Y.L.); hed@lzu.edu.cn (D.H.); 2 Key Laboratory of Veterinary Pharmaceutical Development, Ministry of Agriculture and Rural Affairs, Lanzhou Institute of Husbandry and Pharmaceutical Sciences of Chinese Academy of Agriculture Sciences, No. 335, Qilihe District, Lanzhou 730050, China; zhouds100@163.com (D.Z.); ljs1210173664@163.com (J.L.); lishangwei6636@sina.com (L.T.)

**Keywords:** harmine, synthesis, antibacterial activity, pharmacokinetics

## Abstract

A series of 16 new derivatives of harmine N^9^-Cinnamic acid were synthesized and fully characterized using NMR and MS. The in vitro antibacterial evaluation revealed that most of the synthesized harmine derivatives displayed better antibacterial activities against Gram-positive strains (*S. aureus*, *S. albus* and MRSA) than Gram-negative strains (*E. coli* and PA). In particular, compound **3c** showed the strongest bactericidal activity with a minimum inhibitory concentration of 13.67 μg/mL. MTT assay showed that compound **3c** displayed weaker cytotoxicity than harmine with IC_50_ of 340.30, 94.86 and 161.67 μmol/L against WI-38, MCF-7 and HepG2 cell lines, respectively. The pharmacokinetic study revealed that the distribution and elimination of **3c** in vivo were rapid in rats with an oral bioavailability of 6.9%.

## 1. Introduction

With the increasing use of antibiotics in humans and agriculture, the resistance has spread worldwide. Meanwhile, the decreasing effectiveness of antibiotics in treating common infections has quickened [1,2,3]. After the golden age of the antibiotic discovery between 1929 and the 1970s, the development of new antibiotics has stalled. Since then, few new antibiotics have reached the market [4,5,6,7,8,9]. As a consequence, the development of novel and efficient antibiotics is of great significance.

**Figure 1:**
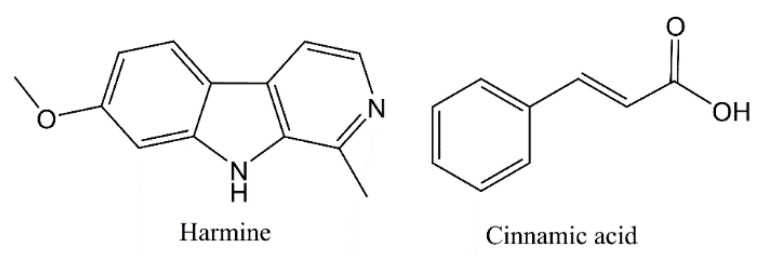
Chemical structure of harmine and cinnamic acid.

Harmine (Figure 1), a naturally existing tricyclic β-carboline alkaloid, has been discovered and isolated from the seeds of *Peganum harmala* L. by Gobel in 1841 [10,11,12]. Harmine displays a wide range of pharmacological activities, including antimicrobial, antitumor, anti-inflammatory, antidepressant, antioxidant, neuroprotective effects and so on [13,14]. It is reported that the extracts of *P. harmala* seeds and roots exhibit good antibacterial activities [15,16]. The literature indicated that harmine and its derivatives insert into DNA of microorganism as a highly aromatic planar alkaloid [11]. At present, harmine derivatives are known for their antibacterial activity and researchers are focusing on studying new antibacterial compounds which are more effective than the drugs now in use [17,18].

Cinnamic acid (Figure 1) is isolated from cinnamon or benzoin resin. Cinnamic acid showed good antibacterial activities in vivo and in vitro [19,20,21,22]. It is reported that cinnamic acid exerted its antibacterial activity mainly by an action mode of membrane disruption [21,23]. Because of its low molecular weight and low price, cinnamic acid is often combined with other compounds to exert antibacterial activity synergistically in the design of new antibacterial drugs [21,24,25]. Using the molecular assembly method, a series novel pleuromutilin derivations bearing cinnamic acids moieties were synthesized which showed remarkable antibacterial activity [25]. Narasimhan and coauthors’ research indicated that the antibacterial activity of synthesized ester derivatives of cinnamic acid was stronger than that of amide [26]. Furthermore, it is reported that the different substituents on the benzene ring of cinnamic acid had an important effect on its antibacterial activity [27].

In this work, based on the combination principle, a series of harmine derivatives with diverse length linkers were synthesized by covalent bonding between N^9^ of harmine and carboxylic acid of cinnamic acids. We further preliminarily investigated the antibacterial activities and cytotoxic activity of the synthesized compounds in vitro. Finally, we performed pharmacokinetics on one of the screened compounds.

## 2. Result and Discussion

### 2.1. Chemistry

**Scheme 1:**
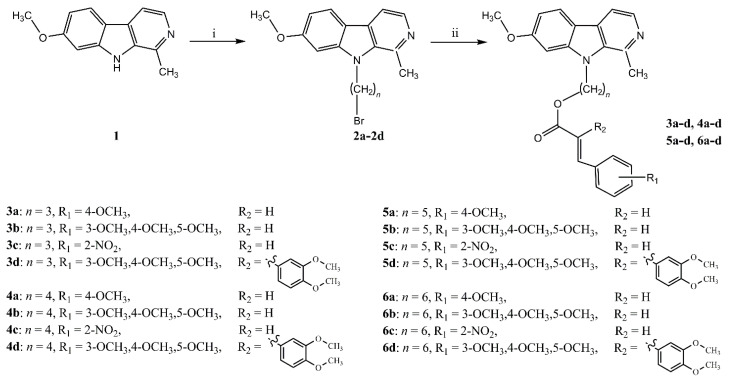
A facile route to the synthesis of target compounds. Reagents and conditions: (i) NaH (1.5 equiv.), Br(CH_2_)*n*Br (3 equiv.), dry DMF, dry THF, reflux, 3 h, 66.1–70.5%; (ii) Substituted cinnamic acid (0.33 equiv.), Et_3_N (5–10 drops), acetone, rt, 3 h, 21.1–52.1%.

The synthesis route of target compounds is shown in Scheme 1. Firstly, in order to explore the effect of chain length on biological activity, dibromoalkanes with different carbon chain lengths used as a electrophilic agent, easily attacked the *N9* position of harmine (**1**) to yield intermediates **2a**–**d**. Subsequently, intermediates **2a**–**d** were combined with substituted cinnamic acid by a nucleophilic substitution reaction to obtain 16 new target compounds (**3a**–**d**, **4a**–**d**, **5a**–**d**, **6a**–**d**).

The structure information of all synthesis compounds were confirmed by nuclear magnetic resonance spectra (NMR), including ^13^C and ^1^H-NMR, and electrospray ionization high-resolution mass spectrometry (ESI-HRMS) (^13^C and ^1^H-NMR spectra of compounds **3a**–**d**, **4a**–**d**, **5a**–**d**, **6a**–**d** had been added in Supplementary Material, Figures S1–S16). Furthermore, a colorless crystal of compound **4a** was obtained by slow evaporation of a mixed solution (ethyl acetate:petroleum ether = 1:4). The crystal structures of compound **4a** are shown in Figure 2. Crystal data for compound **4a** were collected in Tables S1–S3.

### 2.2. Evaluation of the Antibacterial Activity 

According to microplate dilution method, all the prepared compounds were screened for their in vitro antibacterial activity. The investigation included three representative Gram-positive strains, including *Staphylococcus aureus* CICC 10384 (*S. aureus*), *Staphylococcus albus* CICC 20237 (*S. albus*) and *methicillinresistant* *Staphylococcus aureus* ATCC 43300 (MRSA), and two representative Gram-negative strains, including *Escherichia coli* CICC 10389 (*E. coli*) and *Pseudomonas aeruginosa* CVCC 3359 (PA). Harmine and cinnamic acid were used as reference drugs. The results of minimum inhibitory concentration (MICs) are presented in Table 1. It showed that most of the synthesized harmine derivatives displayed better antibacterial activities against Gram-positive strains (*S. aureus*, *S. albus* and MRSA) than Gram-negative strains (*E. coli* and PA). Notably, Compounds **3c** and **5a** displayed attractive antibacterial activities than other derivatives.

**Figure 3:**
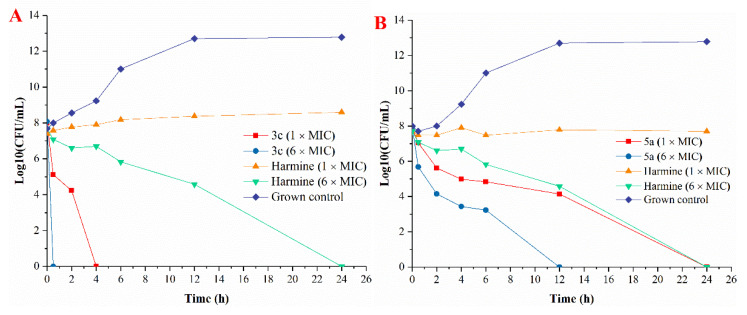
Time-kill kinetics of compound **3c** (**A**) and **5a** (**B**) at 1 × and 6 × MIC against *S. aureus* (CICC 10384).

Because compounds **3c** and **5a** displayed promising antibacterial activity, we further evaluated their in vitro time-kill assay.The bactericidal properties of **3c** and **5a** were compared at 1 × MIC and 6 × MIC against *S. aureus* (the MICs of **3c**, **5a** and harmine were 13.67, 15.63, 31.25 μg/mL, respectively) (Figure 3). As shown in Figure 3, **3c** and **5a** showed outstanding antibacterial activity and displayed a concentration-dependent effect. Compound **3c** with 1 × MIC concentration achieved a 4-log10 reduction in about 4 h and 1 × MIC of compound **5a** showed a 4-log10 reduction in about 24 h. Notably, Compound **3c** with 6 × MIC concentration completely killed the bacteria within the first 0.5 h and 6 × MIC of compound **5a** completely killed the bacteria within 12 h. However, 1 × MIC of harmine just slowed bacterial propagation and its 6 × MIC achieved a 4-log10 reduction in about 24 h. In general, compared to harmine, **3c** and **5a** showed more rapid bactericidal kinetics against *S. aureus* with the same concentration (1 × MIC).

### 2.3. In Vitro Cytotoxic Activity

In vitro cytotoxic activity of **3c** was evaluated using MTT (3-(4,5)-dimethylthi ahiazo (-2)-3,5-diphenytetrazoliiumromide) assay using WI-38 (Human embryonic lung fibroblasts), MCF-7 (human breast cancer cells) and HepG2 (human liver carcinoma cells). Harmine was used as a control. The calculated IC_50_ (half maximal inhibitory concentration) values of **3c** and harmine are presented in Table 2. It was observed that all the tested compounds showed weaker cytotoxic activity against normal cell than cancer cell lines. Notably, the IC_50_ of compound **3c** was higher than that of harmine against three cell lines, which means the cytotoxic activity of compound **3c** was weaker than that of harmine. These combined results suggested that although a nitro group existed in compound **3c**, its cytotoxic activity had not been improved, which showed that the further study of **3c** is meaningful.

### 2.4. In Vivo Pharmacokinetic Study

**Figure 4:**
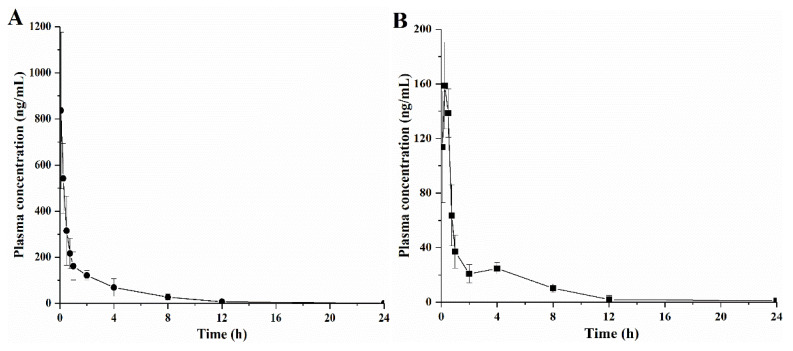
Mean plasma concentration-time profile after a single intravenous administration of 10 mg/kg **3c** solution to rats (**A**) and after a single oral dose of 40 mg/kg **3c** solution (**B**) (*n* = 6, mean ± SE).

Considering the excellent antibacterial activity, we further evaluated the in vivo pharmacokinetics of compound **3c**. The mean plasma concentration time curves of **3c** after intravenous (i.v.) and intragastric (i.g.) administration are shown in Figure 4. Additionally, the estimated non-compartmental parameters are displayed in Table 3.

After a single i.v. and i.g. administration of **3c**, the plasma concentration of the drug declined quickly (Figure 4). After 12 h, the compound **3c** was almost completely eliminated. These data demonstrated that the distribution and elimination of **3c** in vivo were relatively fast. Additionally, compound **3c** displayed great individual differences among animals in vivo. Furthermore, the AUC*0*→*∞* of compound **3c** after a single i.g. administration (290.25 ng·h/mL) was only 6.9% of the same dose of i.v. administration (1053.24 ng·h/mL). These results indicated that compound **3c** had a significant first pass effect, which need to be paid attention in the further research. In addition, the pharmacokinetic data showed that the plasma concentration of **3c** was much lower than its MIC. It reminded us that we should increase the dosage in the follow-up pharmacodynamics study to achieve an effective plasma concentration.

## 3. Materials and Methods

### 3.1. General Information

All chemical reagents were purchased from Energy Reagent Co., Ltd. (Anqing, China) or Shanghai Huangdi Chemical Co., Ltd. (Shanghai, China). Unless otherwise noted, all commercially available reagents were used directly after they were received. In order to monitor the reaction, thin-layer chromatography (TLC) was performed on silica gel plate (Qingdao Haiyang Chemical Co., Ltd., Qingdao, China) and visualized by UV light. All melting points were determined on a digital X4 melting point analyzer (Henan Ruike Medical Instrument Co., Ltd., Xinxiang, China) using open capillary tubes and were uncorrected. HRMS were obtained with a ESI-Brucker APEX Ⅱ49e mass spectrometer (Bruker, Billerica, MA, USA). ^1^H-NMR and ^13^C-NMR spectra were recorded with JNM-ECS (JEOL Co., Ltd., Tokyo, Japan) and Bruke AM-400 spectrometer (Bruker, Billerica, MA, USA), respectively. The solvents used were deuterated reagents (DMSO-*d*_6_ and CDCl_3_). Unless otherwise noted, further purification of products was performed via column chromatography on silica gel (200–300 mesh, Gansu Yihua chemical Glass Instrument Co., Lanzhou, China) and the products were eluted in an appropriate solvent mixture under air pressure. The crystal of the typical compound was obtained by solvent evaporation method and determined by Super Nova single crystal diffractometer (Agilent, Santa Clara, CA, USA).

### 3.2. Synthesis

General procedure for synthesis of compounds **2a**–**d**. To a round-bottom flask 7-methoxy-1-methyl-9*H*-pyridine [3,4-*b*] indole (**1**) (5 mmol) was added in the mixed solvent of DMF and THF (DMF:17 mL, THF:15 mL), stirred at room temperature until clear. NaH (7.5 mmol) was added in batches, stirred for 10 min at room temperature. Then, dibromoalkanes (15 mmol) were added to the mixture, followed by heating and refluxing. The reaction was monitored by TLC during the whole process. After the mixture fully reacted, the mixture was cooled with ice water, and extracted three times with ethyl acetate. The mixture was washed with ether and saturated NaCl solution, after being dried over anhydrous Na_2_SO_4_, the mixture was filtered and concentrated. The crude product was purified by silica gel column to obtain **2a**–**d** (dichloromethane:methanol = 10:1, triethylamine 1‰).

*9-(3-Bromopropyl)-7-methoxy-1-methyl-9H-pyridine [3,4-b] indole* (**2a**). Compound **2a** was prepared according to the general procedure from 7-methoxy-1-methyl-9H-pyridine [3,4-*b*] indole (**1**) and 1,3-dibromopropane. White solid, 68.8% yield. ^1^H-NMR (400 MHz, CDCl_3_) δ: 8.67 (d, *J* = 5.4 Hz, 1H), 8.02 (d, *J* = 8.7 Hz, 1H), 7.87 (d, *J* = 5.4 Hz, 1H), 6.86–6.79 (m, 2H), 4.36 (t, *J* = 7.4 Hz, 2H), 3.91 (s, 3H), 3.51 (t, *J* = 6.4 Hz, 2H), 2.98 (s, 3H), 1.98–1.87 (m, 2H).

*9-(4-Bromobutyl)-7-methoxy-1-methyl-9H-pyridine [3,4-b] indole* (**2b**). Compound **2b** was prepared according to the general procedure from 7-methoxy-1-methyl-9H-pyridine [3,4-*b*] indole (**1**) and 1,4-dibromobutane. White solid, 70.1% yield.^1^H-NMR (400 MHz, CDCl_3_) δ: 8.21 (d, *J* = 5.2 Hz, 1H), 7.98 (d, *J* = 8.6 Hz, 1H), 7.77 (d, *J* = 5.2 Hz, 1H), 6.97–6.80 (m, 2H), 4.54 (t, *J* = 7.5 Hz, 2H), 3.95 (s, 3H), 3.49 (t, *J* = 6.4 Hz, 2H), 3.01 (s, 3H), 1.96–1.81 (m, 4H).

*9-(5-Bromopentyl)-7-methoxy-1-methyl-9H-pyridine [3,4-b] indole* (**2c**). Compound **2c** was prepared according to the general procedure from 7-methoxy-1-methyl-9H-pyridine [3,4-*b*] indole (**1**) and 1,5-dibromopentane. White solid, 68.3% yield. ^1^H-NMR (400 MHz, CDCl_3_) δ: 8.29 (d, *J* = 4.8 Hz, 1H), 7.99 (d, *J* = 8.6 Hz, 1H), 7.74 (d, *J* = 5.4 Hz, 1H), 6.95–6.81 (m, 2H), 4.49 (t, *J* = 7.4 Hz, 2H), 3.96 (s, 3H), 3.41 (t, *J* = 6.4 Hz, 2H), 3.02 (s, 3H), 1.98–1.82 (m, 4H), 1.59–1.44 (m, 2H).

*9-(6-Bromohexyl)-7-methoxy-1-methyl-9H-pyridine [3,4-b] indole* (**2d**). Compound **2d** was prepared according to the general procedure from 7-methoxy-1-methyl-9H-pyridine [3,4-*b*] indole (**1**) and 1,6-dibromohexane. White solid, 66.1% yield. ^1^H-NMR (400 MHz, CDCl_3_) δ: 8.29 (d, *J* = 5.4 Hz, 1H), 7.99 (d, *J* = 8.4 Hz, 1H), 7.74 (d, *J* = 4.0 Hz, 1H), 6.96–6.79 (m, 2H), 4.48 (t, *J* = 7.5 Hz, 2H), 3.96 (s, 3H), 3.40 (t, *J* = 6.4 Hz, 2H), 3.02 (s, 3H), 1.89–1.78 (m, 4H), 1.51–1.39 (m, 4H).

General procedure for synthesis of compounds **3a**–**d**, **4a**–**d**, **5a**–**d**, **6a**–**d**. To a round-bottom flask with a magnetic stirrer substituted cinnamic acid (1 mmol) in acetone (8 mL), triethylamine were added dropwise (5–10 drops), and stirred until completely dissolved. Compound **2a**–**d** (3 mmol) was dissolved and dropped into the above reaction solution, stirred at room temperature for 3 h. The reaction was monitored by TLC during the whole process. After the mixture fully reacted, aqueous HCl was added to the solution to adjust pH to 3–5. The mixture was extracted three times with ethyl acetate. The organic phase was collected, washed twice with saturated NaHCO_3_ solution and saturated NaCl solution, respectively. The organic phase was then dried over anhydrous NaSO_4_, filtered and concentrated. Compounds **3a**–**d**, **4a**–**d**, **5a**–**d**, **6a**–**d** were obtained by further purification using silica gel column chromatography (dichloromethane:methanol = 10:1, triethylamine 1‰).

*3-(7-Methoxy-1-methyl-9H-pyridine [3,4-b] indole-9-yl)propyl(E)-3-(4-methoxyphenyl) acrylate* (**3a**). Compound **3a** was prepared according to the general procedure from *(E*)-3-(4-methoxyphenyl)acrylic acid and 9-(3-bromopropyl)-7-methoxy-1-methyl-9*H*-pyridine [3,4-*b*] indole (**2a**). Yellow solid. 52.1% yield; mp 133.2–133.9 °C; ^1^H-NMR (400 MHz, CDCl_3_) δ: 8.29 (d, *J* = 5.2 Hz, 1H), 7.96 (d, *J* = 8.4 Hz, 1H), 7.73 (d, *J* = 5.2 Hz, 1H), 7.65 (d, *J* = 16.0 Hz, 1H), 7.49 (d, *J* = 8.8 Hz, 2H), 6.94–6.87 (m, 4H), 6.29 (d, *J* = 16.0 Hz, 1H), 4.65 (t, *J* = 7.6 Hz, 2H), 4.28 (t, *J* = 5.8 Hz, 2H), 3.91 (s, 3H), 3.86 (s, 3H), 3.06 (s, 3H), 2.28–2.22 (m, 2H); ^13^C-NMR (100 MHz, CDCl_3_) δ: 166.97, 161.53, 160.89, 144.97, 142.95, 140.47, 138.48, 135.12, 129.76, 129.48, 122.35, 115.21, 114.79, 114.33, 112.22, 108.82, 93.21, 61.42, 55.58, 55.34, 41.79, 29.86, 23.35; ESI-MS: calcd. for C_26_H_26_N_2_O_4_ [M + H]^+^ 431.1965; found 431.1955.

*3-(7-Methoxy-1-methyl-9H-pyridine [3,4-b] indole-9-yl) propyl (E)-3-(3,4,5-trimethoxyphenyl) acrylate* (**3b**). Compound **3b** was prepared according to the general procedure from (*E*)-3-(3,4,5-trimethoxyphenyl) acrylic acid and 9-(3-bromopropyl)-7-methoxy-1-methyl-9*H*-pyridine [3,4-*b*] indole (**2a**). White solid. 49.2% yield; m.p.: 102.6–104.2 °C; ^1^H-NMR (400 MHz, CDCl_3_) δ: 8.29 (d, *J* = 5.2 Hz, 1H), 7.96 (d, *J* = 9.2 Hz, 1H), 7.73 (d, *J* = 5.2 Hz, 1H), 7.58 (d, *J* = 16.0 Hz, 1H), 6.90 (s, 2H), 6.76 (s, 2H), 6.31 (d, *J* = 16.0 Hz, 1H), 4.64 (t, *J* = 7.4 Hz, 2H), 4.29 (t, *J* = 5.8 Hz, 2H), 3.92 (s, 9H), 3.90 (s, 3H), 3.05 (s, 3H), 2.28–2.22 (m, 2H); ^13^C-NMR (100 MHz, CDCl_3_) δ: 166.75, 161.05, 153.56, 145.42, 143.12, 140.54, 140.44, 138.59, 135.28, 129.73, 122.54, 116.68, 115.41, 112.40, 108.90, 105.41, 93.48, 61.80, 61.08, 56.28, 55.77, 41.97, 31.82, 29.95, 29.36, 23.45; ESI-MS: calcd. for C_28_H_30_N_2_O_6_ [M + H]^+^ 491.2177; found 491.2171.

*4-(7-Methoxy-1-methyl-9H-pyridine [3,4-b] indole-9-yl) propyl(E)-3-(2-nitrophenyl) acrylate* (**3c**). Compound **3c** was prepared according to the general procedure from (*E*)-3-(2-nitrophenyl) acrylic acid and 9-(3-bromopropyl)-7-methoxy-1-methyl-9*H*-pyridine [3,4-*b*] indole (**2a**). Yellow solid. 44.2% yield; m.p.: 128.6–129.3 °C; ^1^H-NMR (400 MHz, CDCl_3_) δ: 8.29 (d, *J* = 5.2 Hz, 1H), 8.14 (d, *J* = 16.0 Hz, 1H), 8.08 (d, *J* = 7.2 Hz, 1H), 7.97 (d, *J* = 9.2 Hz, 1H), 7.73–7.64 (m, 3H), 7.60–7.56 (m, 1H), 6.90–6.87 (m, 2H), 6.33 (d, *J* = 16.0 Hz, 1H), 4.67 (t, *J* = 7.4 Hz, 2H), 4.33–4.29 (m, 2H), 3.92 (s, 3H), 3.05 (s, 3H), 2.30–2.24 (m, 2H); ^13^C-NMR (100 MHz, CDCl_3_) δ: 165.67, 160.75, 148.18, 142.94, 140.40, 139.88, 138.10, 135.16, 130.41, 130.19, 129.26, 128.98, 124.79, 123.03, 122.25, 115.09, 112.14, 108.53, 93.31, 64.62, 55.60, 44.66, 28.47, 23.31; ESI-MS: calcd. for C_25_H_23_N_3_O_5_ [M + H]^+^ 446.1710; found 446.1716.

*3-(7-Methoxy-1-methyl-9H-pyridine [3,4-b] indole-9-yl) propyl (E)-2-(3,4-dimethoxyphenyl)-3-(3,4,5-trimethoxyphenyl) acrylate* (**3d**). Compound **3d** was prepared according to the general procedure from (*E*)-2-(3,4-dimethoxyphenyl)-3-(3,4,5-trimethoxyphenyl) acrylic acid and 9-(3-bromopropyl)-7-methoxy-1-methyl-9H-pyridine [3,4-*b*] indole (**2a**). White solid. 21.14% yield; m.p.: 173.6–175.2 °C; ^1^H-NMR (400 MHz, CDCl_3_) δ: 8.28 (d, *J* = 5.2 Hz, 1H), 7.95 (d, *J* = 8.4 Hz, 1H), 7.71 (d, *J* = 5.2 Hz, 1H), 7.67 (s, 1H), 6.95 (d, *J* = 8.4 Hz, 1H), 6.89–6.83 (m, 2H), 6.80 (d, *J* = 9.2 Hz, 2H), 6.36 (s, 2H), 4.56 (t, *J* = 7.6 Hz, 2H), 4.33 (t, *J* = 5.8 Hz, 2H), 3.90 (s, 3H), 3.89 (s, 3H), 3.83 (s, 6H), 3.60 (s, 6H), 2.95 (s, 3H), 2.24–2.19 (m, 2H); ^13^C-NMR (100 MHz, CDCl_3_) δ: 167.73, 161.00, 152.65, 149.46, 148.86, 143.00, 140.64, 140.37, 139.16, 138.38, 135.11, 130.86, 129.63, 128.40, 122.50, 122.23, 115.32, 112.92, 112.33, 111.68, 108.62, 108.17, 93.45, 62.51, 60.88, 55.75, 55.69, 41.99, 29.78, 23.24; ESI-MS: calcd. for C_36_H_38_N_2_O_8_ [M + H]^+^ 627.2701; found 627.2701.

*4-(7-Methoxy-1-methyl-9H-pyridine [3,4-b] indole-9-yl) butyl (E)-3-(4-methoxyphenyl) acrylate* (**4a**). Compound **4a** was prepared according to the general procedure from *(E*)-3-(4-methoxyphenyl) acrylic acid and 9-(4-bromobutyl)-7-methoxy-1-methyl-9*H*-pyridine [3,4-*b*] indole (**2b**). White solid. 43.6% yield; m.p.: 118.7–120.2 °C; ^1^H-NMR (400 MHz, CDCl_3_) δ: 8.29 (d, *J* = 5.2 Hz, 1H), 7.98 (d, *J* = 9.6 Hz, 1H), 7.74 (d, *J* = 5.2 Hz, 1H), 7.63 (d, *J* = 16.0 Hz, 1H), 7.46 (d, *J* = 8.8 Hz, 2H), 6.91–6.88 (m, 4H), 6.29 (d, *J* = 16.0 Hz, 1H), 4.54 (t, *J* = 7.6 Hz, 2H), 4.25 (t, *J* = 6.4 Hz, 2H), 3.94 (s, 3H), 3.84 (s, 3H), 3.03 (s, 3H), 2.01–1.94 (m, 2H), 1.867–1.80 (m, 2H); ^13^C-NMR (100 MHz, CDCl_3_) δ: 167.21, 161.44, 160.88, 144.76, 142.95, 140.44, 138.38, 135.21, 129.75, 129.42, 126.95, 122.39, 115.23, 115.12, 114.30, 112.26, 108.66, 93.37, 63.47, 55.68, 55.67, 44.38, 27.18, 26.13, 23.41; ESI-MS: calcd. for C_27_H_28_N_2_O_4_ [M + H]^+^ 445.2111; found 445.2116.

*4-(7-Methoxy-1-methyl-9H-pyridine [3,4-b] indole-9-yl) butyl (E)-3-(3,4,5-trimethoxyphenyl) acrylate* (**4b**). Compound **4b** was prepared according to the general procedure from (*E*)-3- (3,4,5-trimethoxyphenyl) acrylic acid and 9-(4-bromobutyl)-7-methoxy-1-methyl-9*H*-pyridine [3,4-*b*] indole (**2b**). Yellow solid. 49.0% yield; m.p.: 95.7–96.3 °C; ^1^H-NMR (400 MHz, CDCl_3_) δ: 8.29 (d, *J* = 5.2 Hz, 1H), 7.99 (d, *J* = 9.2 Hz, 1H), 7.75 (d, *J* = 5.2 Hz, 1H), 7.59 (d, *J* = 16.0 Hz, 1H), 6.91–6.89 (m, 2H), 6.74 (s, 2H), 6.31 (d, *J* = 16.0 Hz, 1H), 4.55 (t, *J* = 7.8 Hz, 2H), 4.26 (t, *J* = 6.2 Hz, 2H), 3.94 (s, 3H), 3.89 (s, 9H), 3.03 (s, 3H), 2.02–1.95 (m, 2H), 1.87–1.80 (m, 2H); ^13^C-NMR (100 MHz, CDCl_3_) δ: 166.83, 160.87, 153.40, 145.08, 142.95, 140.41, 140.19, 138.40, 135.21, 129.69, 129.44, 122.42, 116.87, 115.26, 112.28, 108.61, 105.23, 93.43, 63.67, 60.93, 56.13, 55.69, 44.37, 27.19, 26.12, 23.42; ESI-MS: calcd. for C_29_H_32_N_2_O_6_ [M + H]^+^ 505.2333; found 505.2339.

*4-(7-Methoxy-1-methyl-9H-pyridine [3,4-b] indole-9-yl) butyl (E)-3-(2-nitrophenyl) acrylate* (**4c**). Compound **4c** was prepared according to the general procedure from (*E*)-3-(2-nitrophenyl) acrylic acid and 9-(4-bromobutyl)-7-methoxy-1-methyl-9*H*-pyridine [3,4-*b*] indole (**2b**). White solid. 43.6% yield; m.p.: 130.9–132.2 °C; ^1^H-NMR (400 MHz, CDCl_3_) δ: 8.28 (d, *J* = 5.2 Hz, 1H), 8.11 (d, *J* = 15.6 Hz, 1H), 8.04 (d, *J* = 8.0 Hz, 1H), 7.97 (d, *J* = 9.2 Hz, 1H), 7.73 (d, *J* = 5.2 Hz, 1H), 7.67–7.60 (m, 2H), 7.56–7.52(m, 1H), 6.90–6.87 (m, 2H), 6.34 (d, *J* = 16.0 Hz, 1H), 4.54 (t, *J* =7.8 Hz, 2H), 4.27 (t, *J* = 6.4 Hz, 2H), 3.94 (s, 3H), 3.03 (s, 3H), 2.01–1.93 (m, 2H), 1.87–1.80 (m, 2H); ^13^C-NMR (100 MHz, CDCl_3_) δ: 165.62, 160.88, 148.22, 142.94, 140.41, 140.38, 138.36, 135.17, 133.49, 130.40, 130.33, 129.43, 129.08, 124.88, 122.73, 122.37, 115.20, 112.24, 108.69, 93.34, 64.07, 55.67, 44.32, 27.16, 26.03, 23.38; ESI-MS: calcd. for C_26_H_25_N_3_O_5_ [M + H]^+^ 460.1867; found 460.1865.

*4-(7-Methoxy-1-methyl-9H-pyridine [3,4-b] indole-9-yl) butyl (E)-2-(3,4-dimethoxyphenyl)-3-(3,4,5-trimethoxyphenyl) acrylate* (**4d**). Compound **4d** was prepared according to the general procedure from (*E*)-2-(3,4-dimethoxyphenyl)-3-(3,4,5-trimethoxyphenyl) acrylic acid and 9-(4-bromobutyl)-7-methoxy-1-methyl-9*H*-pyridine [3,4-*b*] indole (**2b**). White solid. 27.2% yield; m.p.: 95.7–97.4 °C; ^1^H-NMR (400 MHz, CDCl_3_) δ: 8.29 (d, *J* = 5.6 Hz, 1H), 7.98 (d, *J* = 8.8 Hz, 1H), 7.73 (d, *J* = 5.2 Hz, 1H), 7.69 (s, 1H), 6.89 (dd, *J* = 2.0, 8.8 Hz, 1H), 6.83–6.81 (m, 2H), 6.76 (dd, *J* = 2.0, 8.4 Hz, 1H), 6.71 (d, *J* = 2.0 Hz, 1H), 6.34 (s, 2H), 4.47–4.43 (m, 2H), 4.29 (t, *J* = 6.2 Hz, 2H), 3.92 (s, 3H), 3.81 (s, 3H), 3.80 (s, 3H), 3.70 (s, 3H), 3.56 (s, 6H), 2.99 (s, 3H), 1.94–1.89 (m, 2H), 1.87–1.78 (m, 2H); ^13^C-NMR (100 MHz, CDCl_3_) δ: 167.84, 161.86, 152.62, 149.30, 148.72, 144.00, 140.48, 139.07, 135.45, 134.70, 131.03, 129.65, 128.42, 122.97, 122.18, 119.25, 114.68, 112.86, 111.54, 109.93, 108.11, 105.32, 93.29, 64.26, 60.86, 55.78, 55.72, 44.52, 27.42, 26.18, 21.46; ESI-MS: calcd. for C_37_H_40_N_2_O_8_ [M + H]^+^ 641.2857; found 641.2844.

*5-(7-Methoxy-1-methyl-9H-pyridine [3,4-b] indole-9-yl) amyl (E)-3-(4-methoxyphenyl) acrylate* (**5a**). Compound **5a** was prepared according to the general procedure from *(E*)-3-(4-methoxyphenyl) acrylic acid and 9-(5-bromopentyl)-7-methoxy-1-methyl-9*H*-pyridine [3,4-*b*] indole (**2c**). White solid. 46.2% yield; m.p.: 91.1–92.3 °C; ^1^H-NMR (400 MHz, DMSO-*d*_6_) δ: 8.17 (d, *J* = 5.2 Hz, 1H), 8.08 (d, *J* = 8.4 Hz, 1H), 7.88 (d, *J* = 5.2 Hz, 1H), 7.66 (d, *J* = 8.8 Hz, 2H), 7.57 (d, *J* = 16.0 Hz, 1H), 7.20 (s, 1H), 6.98 (d, *J* = 8.4 Hz, 2H), 6.87 (d, *J* = 8.6 Hz, 1H), 6.44 (d, *J* = 16.0 Hz, 1H), 4.57 (t, *J* = 7.6 Hz, 2H), 4.13 (t, *J* = 6.6 Hz, 2H), 3.91 (s, 3H), 3.81 (s, 3H), 2.96 (s, 3H), 1.82–1.74 (m, 2H), 1.72–1.69 (m, 2H); 1.51–1.43 (m, 2H); ^13^C-NMR (100 MHz, CDCl_3_) δ: 167.22, 161.33, 160.84, 144.44, 143.00, 140.31, 138.03, 135.15, 129.66, 129.40, 126.99, 122.33, 115.31, 115.10, 114.24, 112.21, 108.64, 93.32, 63.79, 55.63, 55.30, 44.66, 30.23, 28.50, 23.38, 23.20; ESI-MS: calcd. for C_28_H_30_N_2_O_4_ [M + H]^+^ 459.2278; found 459.2288.

*5-(7-Methoxy-1-methyl-9H-pyridine [3,4-b] indole-9-yl) amyl (E)-3-(3,4,5-trimethoxyphenyl) acrylate* (**5b**). Compound **5b** was prepared according to the procedure from (*E*)-3-(3,4,5-trimethoxyphenyl) acrylic acid and 9-(5-bromopentyl)-7-methoxy-1-methyl-9H-pyridine [3,4-*b*] indole (**2c**). White solid. 48.9% yield; m.p.: 103.7–104.7 °C; ^1^H-NMR (400 MHz, DMSO-*d*_6_) δ: 8.16 (d, *J* = 5.1 Hz, 1H), 8.08 (d, *J* = 8.4 Hz, 1H), 7.87 (d, *J* = 5.4 Hz, 1H), 7.57 (d, *J* = 15.9 Hz, 1H), 7.20 (s, 1H), 7.07 (s, 2H), 6.86 (d, *J* = 8.7 Hz, 1H), 6.64 (d, *J* = 15.9 Hz, 1H), 4.56 (t, *J* = 7.4 Hz, 2H), 4.14 (t, *J* = 6.5 Hz, 2H), 3.91 (s, 3H), 3.83 (s, 6H), 3.70 (s, 3H), 2.95 (s, 3H), 1.81–1.69 (m, 4H), 1.53–1.44 (m, 2H); ^13^C-NMR (100 MHz, DMSO-*d*_6_) δ: 166.88, 160.99, 153.55, 145.16, 143.21, 140.97, 139.93, 138.19, 135.02, 130.08, 128.90, 122.81, 117.76, 114.68, 112.67, 109.54, 106.40, 94.12, 64.19, 60.56, 56.50, 56.04, 44.41, 30.39, 28.47, 23.53, 23.20; ESI-MS: calcd. for C_30_H_34_N_2_O_6_ [M + H]^+^ 519.2490; found 519.2474.

*5-(7-Methoxy-1-methyl-9H-pyridine [3,4-b] indole-9-yl) amyl (E)-3-(2-nitrophenyl) acrylate* (**5c**). Compound **5c** was prepared according to the procedure from (*E*)-3-(2-nitrophenyl) acrylic acid and 9-(5-bromopentyl)-7-methoxy-1-methyl-9*H*-pyridine [3,4-*b*] indole (**2c**). Yellow solid. 44.2% yield; m.p.: 127.4–130.0 °C; ^1^H-NMR (400 MHz, CDCl_3_) δ: 8.28 (d, *J* = 5.2 Hz, 1H), 8.11 (d, *J* = 16.0 Hz, 1H), 8.06 (d, *J* = 8.0 Hz, 1H), 7.97 (d, *J* = 9.2 Hz, 1H), 7.73 (d, *J* = 5.6 Hz, 1H), 7.69–7.62 (m, 2H), 7.54–7.58 (m, 1H), 6.89–6.86 (m, 2H), 6.34 (d, *J* = 16.0 Hz, 1H), 4.53–4.49 (m, 2H), 4.24 (t, *J* = 6.4 Hz, 2H), 3.95 (s, 3H), 3.03 (s, 3H), 1.95–1.87 (m, 2H), 1.82–1.75 (m, 2H), 1.59–1.50 (m, 2H); ^13^C-NMR (100 MHz, CDCl_3_) δ: 165.82, 158.08, 148.23, 144.12, 140.01, 138.76, 136.94, 133.49, 130.58, 130.22, 129.14, 127.31, 124.87, 123.16, 118.24, 115.39, 113.62, 108.74, 104.15, 99.94, 64.73, 62.57, 59.60, 33.71, 32.46, 31.74, 24.40; ESI-MS: calcd. for C_27_H_27_N_3_O_5_ [M + H]^+^ 474.2023; found 474.2011.

*5-(7-Methoxy-1-methyl-9H-pyridine [3,4-b] indole-9-yl) pentyl (E)-2-(3,4-dimethoxyphenyl)-3- (3,4,5-trimethoxyphenyl) acrylate* (**5d**). Compound **5d** was prepared according to the procedure from (*E*)-2-(3,4-dimethoxyphenyl)-3-(3,4,5-trimethoxyphenyl) acrylic acid and 9-(5-bromopentyl)-7-methoxy-1-methyl-9*H*-pyridine [3,4-*b*] indole (**2c**). White solid. 28.2% yield; m.p.: 117.4–119.9 °C; ^1^H-NMR (400 MHz, CDCl_3_) δ: 8.28 (d, *J* = 5.2 Hz, 1H), 7.97 (d, *J* = 8.8 Hz, 1H), 7.73 (d, *J* = 5.2 Hz, 1H), 7.67 (s, 1H), 6.89 (d, *J* = 2.0 Hz, 1H), 6.87 (s, 1H), 6.85 (s, 1H), 6.77–6.75 (m, 2H), 6.35 (s, 2H), 4.39 (t, *J* = 7.8 Hz, 2H), 4.22 (t, *J* = 6.4 Hz, 2H), 3.94 (s, 3H), 3.86 (s, 3H), 3.82 (s, 3H), 3.79 (s, 3H), 3.58 (s, 6H), 2.96 (s, 3H), 1.88–1.84 (m, 2H), 1.76–1.73 (m, 2H), 1.51–1.43 (m, 2H); ^13^C-NMR (100 MHz, CDCl_3_) δ: 167.69, 162.39, 160.81, 152.47, 149.15, 148.54, 142.98, 140.20, 139.91, 138.86, 137.94, 135.10, 135.09, 131.21, 129.69, 129.37, 128.42, 122.14, 115.02, 112.95, 112.87, 112.15, 111.33, 108.54, 107.92, 93.33, 64.53, 60.72, 55.86, 55.58, 44.60, 30.08, 28.33, 23.26, 23.26, 23.08; ESI-MS: calcd. for C_38_H_42_N_2_O_8_ [M + H]^+^ 665.3014; found 655.3018.

*6-(7-Methoxy-1-methyl-9H-pyridine [3,4-b] indole-9-yl) hexyl (E)-3-(4-methoxyphenyl) acrylate* (**6a**). Compound **6a** was prepared according to the general procedure from *(E*)-3-(4-methoxyphenyl) acrylic acid and 9-(6-bromohexyl)-7-methoxy-1-methyl-9*H*-pyridine [3,4-*b*] indole (**2d**). Yellow solid. 37.6% yield; m.p.: 88.7–90.4 °C; ^1^H-NMR (400 MHz, CDCl_3_) δ: 8.29 (d, *J* = 5.4 Hz, 1H), 7.97 (d, *J* = 8.4 Hz, 1H), 7.73 (d, *J* = 5.1 Hz, 1H), 7.63 (d, *J* = 16.2 Hz, 1H), 7.46 (d, *J* = 8.5 Hz, 2H), 6.84–6.90(m, 4H), 6.29 (d, *J* = 15.9 Hz, 1H), 4.45 (t, *J* = 7.8 Hz, 2H), 4.18 (t, *J* = 6.7 Hz, 2H), 3.94 (s, 3H), 3.82 (s, 3H), 3.02 (s, 3H), 1.90–1.81 (m, 2H), 1.74–1.66 (m, 2H), 1.50–1.45 (m, 4H); ^13^C-NMR (100 MHz, CDCl_3_) δ: 167.25, 161.27, 160.76, 144.30, 142.95, 140.37, 138.08, 135.16, 129.60, 129.28, 127.00, 122.28, 115.42, 115.11, 114.21, 112.15, 108.51, 93.31, 64.03, 55.60, 55.26, 44.68, 30.47, 28.60, 26.52, 25.76, 23.30; ESI-MS: calcd. for C_29_H_32_N_2_O_4_ [M + H]^+^ 473.2435; found 473.2433.

*6-(7-Methoxy-1-methyl-9H-pyridine [3,4-b] indole-9-yl) hexyl (E)-3-(3,4,5-trimethoxyphenyl) acrylate* (**6b**). Compound **6b** was prepared according to the general procedure from (*E*)-3-(3,4,5-trimethoxyphenyl) acrylic acid and 9-(6-bromohexyl)-7-methoxy-1-methyl-9*H*-pyridine [3,4-*b*] indole (**2d**). Yellow solid. 38.1% yield; m.p.: 73.7–73.9 °C; ^1^H-NMR (400 MHz, CDCl_3_) δ: 8.29 (d, *J* = 5.2 Hz, 1H), 7.98 (d, *J* = 8.4 Hz, 1H), 7.74 (d, *J* = 5.2 Hz, 1H), 7.59 (d, *J* = 15.6 Hz, 1H), 6.91–6.86 (m, 2H), 6.75 (s, 2H), 6.33 (d, *J* = 16.0 Hz, 1H), 4.48 (t, *J* = 7.8 Hz, 2H), 4.20 (t, *J* = 6.6 Hz, 2H), 3.95 (s, 3H), 3.88 (s, 9H), 3.02 (s, 3H), 1.90–1.83 (m, 2H), 1.74–1.69 (m, 2H), 1.50–1.47 (m, 4H); ^13^C-NMR (100 MHz, CDCl_3_) δ: 166.95, 160.86, 153.36, 144.70, 143.08, 140.30, 140.05, 137.94, 135.19, 129.81, 129.46, 122.40, 117.21, 115.15, 112.26, 108.57, 105.15, 93.44, 64.24, 60.91, 56.10, 55.68, 44.76, 30.50, 28.61, 26.55, 25.77, 23.19; ESI-MS: calcd. for C_31_H_36_N_2_O_6_ [M + H]^+^ 533.2646; found 533.2648.

*6-(7-Methoxy-1-methyl-9H-pyridine [3,4-b] indole-9-yl) hexyl(E)-3-(2-nitrophenyl) acrylate* (**6c**). Compound **6c** was prepared according to the general procedure from (*E*)-3-(2-nitrophenyl) acrylic acid and 9-(6-bromohexyl)-7-methoxy-1-methyl-9*H*-pyridine [3,4-*b*] indole (**2d**). White solid. 33.9% yield; m.p.: 117.3–119.9 °C; ^1^H-NMR (400 MHz, DMSO-*d*_6_) δ: 8.16 (d, *J* = 5.1 Hz, 1H), 8.08 (d, *J* = 8.7 Hz, 2H), 7.95–7.90 (m, 2H), 7.87 (d, *J* = 5.1 Hz, 1H), 7.78 (t, *J* = 7.5 Hz, 1H), 7.68 (t, *J* = 7.7 Hz, 1H), 7.18 (s, 1H), 6.86 (d, *J* = 8.7 Hz, 1H), 6.62 (d, *J* = 15.9 Hz, 1H), 4.55 (t, *J* = 7.4 Hz, 2H), 4.16 (t, *J* = 6.5 Hz, 2H), 3.90 (s, 3H), 2.95 (s, 3H), 1.78–1.71 (m, 2H), 1.66–1.60 (m, 2H), 1.45–1.40 (m, 4H); ^13^C-NMR (100 MHz, CDCl_3_) δ: 165.67, 160.75, 148.18, 142.94, 140.40, 139.88, 138.10, 135.16, 133.40, 130.41, 130.19, 129.26, 128.98, 124.79, 123.03, 122.25, 115.09, 112.14, 108.53, 93.31, 64.62, 55.60, 44.66, 30.48, 28.47, 26.51, 25.76, 23.31; ESI-MS: calcd. for C_28_H_29_N_3_O_5_ [M + H]^+^ 488.2180; found 488.2201.

*6-(7-Methoxy-1-methyl-9H-pyridine [3,4-b] indole-9-yl) hexyl (E)-2-(3,4-dimethoxyphenyl)-3– (3,4,5-trimethoxyphenyl) acrylate* (**6d**). Compound **6d** was prepared according to the general procedure from (*E*)-2-(3,4-dimethoxyphenyl)-3-(3,4,5-trimethoxyphenyl) acrylic acid and 9-(6-bromohexyl)-7-methoxy-1-methyl-9*H*-pyridine [3,4-*b*] indole (**2d**). White solid. 27.3% yield; m.p.: 113.1–115.0 °C; ^1^H-NMR (400 MHz, CDCl_3_) δ: 8.27 (d, *J* = 5.6 Hz, 1H), 7.99 (d, *J* = 8.8 Hz, 1H), 7.82 (d, *J* = 5.2 Hz, 1H), 7.68 (s, 1H), 6.93 (d, *J* = 8.8 Hz, 1H), 6.84 (s, 2H), 6.79–6.74 (m, 2H), 6.34 (s, 2H), 4.43 (t, *J* = 7.6 Hz, 2H), 4.18 (t, *J* = 6.6 Hz, 2H), 3.95 (s, 3H), 3.81 (s, 3H), 3.79 (s, 3H), 3.77 (s, 3H), 3.55 (s, 6H), 3.11 (s, 3H), 1.83–1.78 (m, 2H), 1.70–1.65 (m, 2H), 1.45–1.36 (m, 4H); ^13^C-NMR (100 MHz, CDCl_3_) δ: 167.90, 161.62, 153.13, 152.62, 149.25, 148.67, 143.88, 140.03, 138.95, 134.89, 131.39, 129.83, 128.61, 122.86, 122.28, 114.83, 113.03, 112.62, 111.47, 109.55, 108.04, 105.24, 93.42, 64.84, 60.86, 55.95, 55.72, 44.85, 30.50, 28.50, 26.46, 25.67; ESI-MS: calcd. for C_39_H_44_N_2_O_8_ [M + H]^+^ 669.3170; found 669.3196.

### 3.3. Evaluation of the Antibacterial Activity

#### 3.3.1. MIC Testing

The antibacterial efficiency of all prepared compounds was determined using the microplate dilution method [28,29]. All compounds were prepared into different concentrations of stock solution according to their solubility. The compounds were added to the 96 well plate and serially diluted in Mueller-Hinton broth. Three Gram-positive strains (*S. aureus*, *S. albus* and MRSA) and two Gram-negative strains (*E. coli* and PA) were cultivated and added to the 96 well plate. The initial concentration of bacteria should be higher than 10^5^ CFU/mL. The samples were incubated at 37 °C for 18–24 h. The MIC was determined for compounds by observing change of clarity in the broth. The same procedure was repeated in triplicate.

#### 3.3.2. Bactericidal Time–Kill Kinetics

The bacteria *S. aureus* was prepared in Muellere Hinton broth at 37 °C for 6 h with shaking. The solution of **3c** (1 × MIC and 6 × MIC), **5a** (1 × MIC and 6 × MIC), harmine (1 × MIC and 6 × MIC) and saline (served as a control group) were separately added to the bacterial suspension so that the final bacterial concentration were 10^6^~10^7^ CFU/mL. At the predetermined time point (0, 0.5, 2, 4, 6, 12 and 24 h), 20 mL of aliquots were serially diluted in normal saline, plated on sterile Muellere Hinton agar plates, and incubated at 37 °C for 24 h. After incubation, the viable colonies were counted and represented as log_10_ (CFU/mL). The same procedure was repeated in triplicate.

### 3.4. In Vitro Cytotoxic Activity

The in vitro cytotoxicity of **3c** was determined using MTT assay. WI-38 (Human embryonic lung fibroblasts), MCF-7 (Human breast cancer cells) and HepG2 (human liver carcinoma cells) were purchased from American Type Culture Collection (Manassas, VA, USA). The cells were maintained using Dulbecco’s modified eagle’s medium (DMEM, Sangon Biotech, Shanghai, China) supplemented with 10% fetal bovine serum (FBS, Sijiqing Bioengineering Material Co., Ltd., Hangzhou, China), 100 U/mL penicillin and 100 μg/mL streptomycin in a humidified 37 °C incubator under 5% CO_2_. 100 μL culture medium at a concentration of 1 × 10^4^ cells/mL was added to 96-well plate and incubated in a humidified 37 °C incubator under 5% CO_2_. After 24 h, 10 µL solution of **3c** and harmine dissolved in DMSO and PBS and then diluted with DMEM under a concentration gradient, which was added in the wells for final concentrations of 1000, 500, 250, 125, 62.5 µmol/L of compounds in WI-38 and 160, 80, 40, 20, 10 µmol/L of compounds in MCF-7 and Hep-G2. After 48 h incubation, 10 μL MTT solution (5 mg/mL) was added into each well and incubated in a humidified 37 °C incubator under 5% CO_2_. After 4 h, the supernatant was removed and 150 μL DMSO was added. The absorbance of each well was determined by an enzyme-linked immunosorbent assay reader (Thermo Fisher Scientific Inc., Rochester, NY, USA) at wavelengths of 570 nm. The experiments were performed in triplicate.

### 3.5. In Vivo Pharmacokinetic Study

#### 3.5.1. Animals and Ethics Statement

Adult males and females Sprague Dawley rats, with average weight of 200–250 g, were purchased from the Experiment Animal Center of Lanzhou University (Lanzhou, China). The rats were housed under an environmentally controlled room for one week before staring the experiment. All procedures were performed according to the guidelines issued by the Ethical Committee of Lanzhou University (Permit Number: SYXK(Gan)-2018-0002).

#### 3.5.2. Pharmacokinetic Experiment

After 7 days acclimatization, rats were randomly divided into two groups with half male and half female in each group (*n* = 6). All animals were fasted for 12 h with access to water prior to dosing. One group was intravenously administered with **3c** at dose of 10 mg/kg. Another group was oral administered with **3c** at dose of 40 mg/kg. Approximately 0.3 mL of blood samples were collected into heparinized tubes from the orbital vein at 0.083, 0.25, 0.5, 0.75, 1, 2, 4, 8, 12 and 24 h after the administration. The heparinized blood samples were immediately centrifuged at 4000 rpm for 10 min and the upper plasma was separated and immediately stored at −20 °C used for UPLC-MS/MS analysis.

Plasma samples were processed as follows. First, 100 μL of plasma samples and 50 μL of tinidazole (1000 ng/mL, internal standard) were mixed by vortex for 1 min. Next, 250 μL of acetonitrile were added to the mixture and vortexed for 10 min. After centrifugation at 10,000 rpm for 10 min, 200 μL of the upper organic layer were removed into 1.5 mL centrifuge tube and dried with nitrogen. Finally, the residue was reconstituted in 100 μL of 50% acetonitrile, vortexed for 1 min, centrifuged at 10,000 rpm for 10 min, and then the supernatant was used for UPLC-MS/MS analysis.

#### 3.5.3. Statistical Analysis

The obtained data were analyzed using a non-compartment open model with PKsolver Software [30,31]. Pharmacokinetic parameters and the experiment data were expressed as the mean ± standard deviation (SD). A paired student’s *t*-test was used for statistical analysis, with *p* < 0.05 considered the minimum level of significance.

## 4. Conclusions

In summary, 16 novel harmine derivatives were synthesized by covalent bonding between N^9^ of harmine and carboxylic acid of cinnamic acid derivatives. Most of the synthesized harmine derivatives displayed better antibacterial activities against Gram-positive strains (*S. aureus*, *S. albus* and MRSA) than Gram-negative strains (*E. coli* and PA) in vitro. Notably, compound **3c**, the most effective compound, showed excellent bactericidal activity against *S. aureus* and *S. albus* in time–kill assay. In MTT assay, it showed that compound **3c** had weaker cytotoxicity than harmine with IC_50_ of 340.30, 94.86 and 161.67 μmol/L against WI-38, MCF-7 and HepG2 cell lines, respectively. Pharmacokinetic studies showed that compound **3c** showed rapid absorption and excretion with an oral bioavailability of 6.9% in vivo.

## Figures and Tables

**Figure 2:**
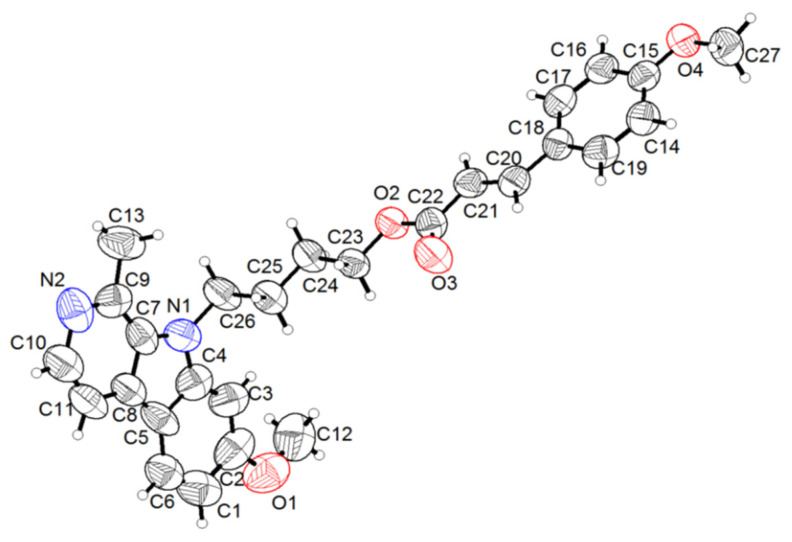
ORTEP diagram for compound **4a** with ellipsoids set at 75% probability.

**Table 1:** Minimum inhibitory concentration of the synthesized harmine derivatives.

Compound	MIC (μg/mL)				
	Gram-Positive Bacteria			Gram-Negative Bacteria	
	*S. aureus*	*S*. *albus*	MRSA	*E*. *coli*	PA
**3a**	325	650	1300	650	325
**3b**	318.75	637.5	637.5	637.5	318.75
**3c**	13.67	13.67	54.69	218.75	218.75
**3d**	181.25	725	362.5	725	362.5
**4a**	305	305	610	152.5	305
**4b**	112.5	225	450	450	450
**4c**	1550	1550	1550	775	387.5
**4d**	26.56	53.13	106.25	212.5	212.5
**5a**	15.63	15.63	62.5	250	125
**5b**	1138	2275	2275	1137.5	1137.5
**5c**	110	110	220	220	220
**5d**	23.83	95.3	95.31	762.5	381.25
**6a**	82.81	165.63	331.25	662.5	331.25
**6b**	152.5	152.5	305.5	305	305
**6c**	375	750	1500	750	750
**6d**	53.13	53.13	212.5	212.5	212.5
Harmine	31.25	15.63	62.50	15.63	15.63
Cinnamic acid	1125	1125	1125	1125	1125
DMSO	-	-	-	-	-

**Table 2:** Inhibition effect of target compounds in three cell lines. (*n* = 3; Mean ± SD).

Compound	IC_50_ (µmol/L)		
	WI-38	MCF-7	HepG2
**3c**	340.30 ± 2.79	94.86 ± 3.64	161.67 ± 1.31
Harmine	103.40 ± 0.96	78.88 ± 2.82	67.61 ±1.60

**Table 3:** The non-compartmental model pharmacokinetic parameters in rats after a single intravenous injection (10 mg/kg) and intragastric administration (40 mg/kg) of compound **3c**. (*n* = 6; Mean ± SD).

Pharmacokinetics Parameters	i.v.	i.g.
*C*max (ng/mL)	836.67 ± 229.45	158.73 ± 59.61
*Tmax* (h)	0.083 ± 0.030	0.25 ± 0.033
*T*1/2 (h)	2.27 ± 0.62	1.30 ± 0.47
*Cl/F* (L/h·kg)	0.031 ± 0.03	0.26 ± 0.10
*MRT* (h)	2.8585 ± 0.71	3.3322 ± 0.83
*AUC0→t* (ng·h/mL)	1050.59 ± 229.31	284.24 ± 54.36
*AUC0→∞* (ng·h/mL)	1053.24 ± 231.06	290.25 ± 55.17

*C*max, maximum concentration; *Tmax*, time to reach *C*max; *T*1/2, half-life; *Cl/F*, Clearance rate; *MRT,* mean resident time; *AUC0→t*, Area under the curve from zero to the last measurable plasma concentration; *AUC0→∞*, Area under the curve, from the last measurable plasma concentration to infinity.

## Data Availability

The original contributions presented in the study are included in the article and Supplementary Material, further inquiries can be directed to the corresponding author.

## Supplementary Materials

The following are available online at, Figures S1–S16: The ^1^H-NMR and ^13^C-NMR spectrum of compound **3a**–**d**, **4a**–**d**, **5a**–**d** and **6a**–**d**; Tables S1–S3: Crystal data of compound **4a**.

## Sample Availability

Samples of the compounds are available from the authors.
