# Supplementary material for: Synthesis, Antibacterial and Pharmacokinetic Evaluation of Novel Derivatives of Harmine N9-Cinnamic Acid
Source: Molecules. 2021 Aug 10;26(16):4842. doi: 10.3390/molecules26164842 (PMC8400480; doi:10.3390/molecules26164842)
Supplement: Supplementary file 1 [file molecules-26-04842-s001.zip › molecules-1310597-supplementary.pdf]

molecules

Supplementary Material

Synthesis, Antibacterial and Pharmacokinetic Evaluation of Novel Derivatives of Harmine N^{9}-Cinnamic Acid

Yan Liang [1], Dian He [1], Deshun Zhou [2], Junshuai Li [2], Lei Tang [2] and Zhen Wang [1,*]

[1] School of Pharmacy, Lanzhou University, West Donggang Road No. 199, Lanzhou 730000, China; liangy18@lzu.edu.cn (Y.L.); hed@lzu.edu.cn (D.H.)
[2] Key Laboratory of Veterinary Pharmaceutical Development, Ministry of Agriculture and Rural Affairs, Lanzhou Institute of Husbandry and Pharmaceutical Sciences of Chinese Academy of Agriculture Sciences, No. 335, Qilihe District, Lanzhou 730050, China; zhouds100@163.com (D.Z.); ljs1210173664@163.com (J.L.); lishangwei6636@sina.com (L.T.)
* Correspondence: liangjp100@impcas.ac.cn Tel.: +86-0931-2115287

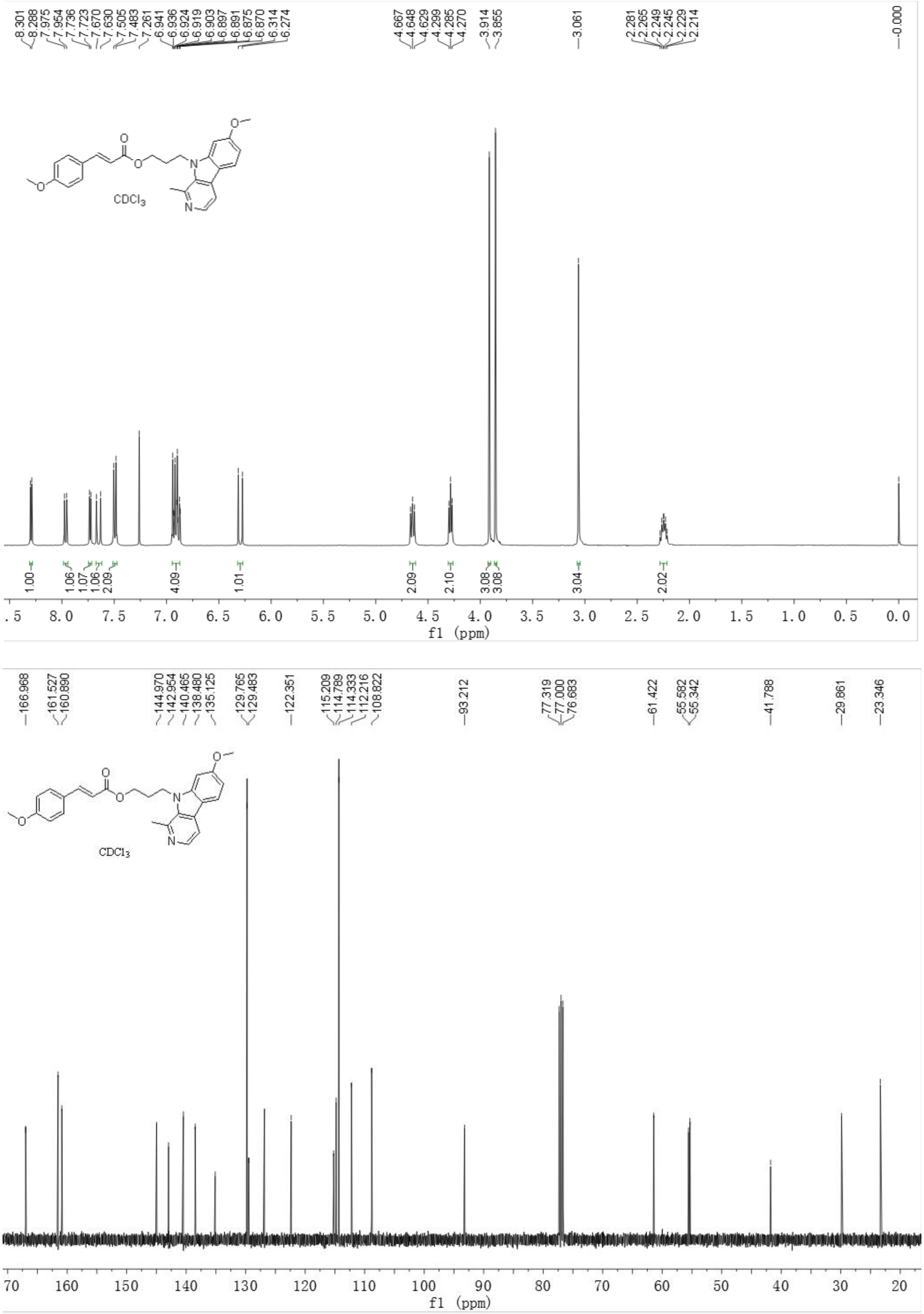

Figure S1. The ^{1}H-NMR and ^{13}C-NMR spectrum of compound **3a**.

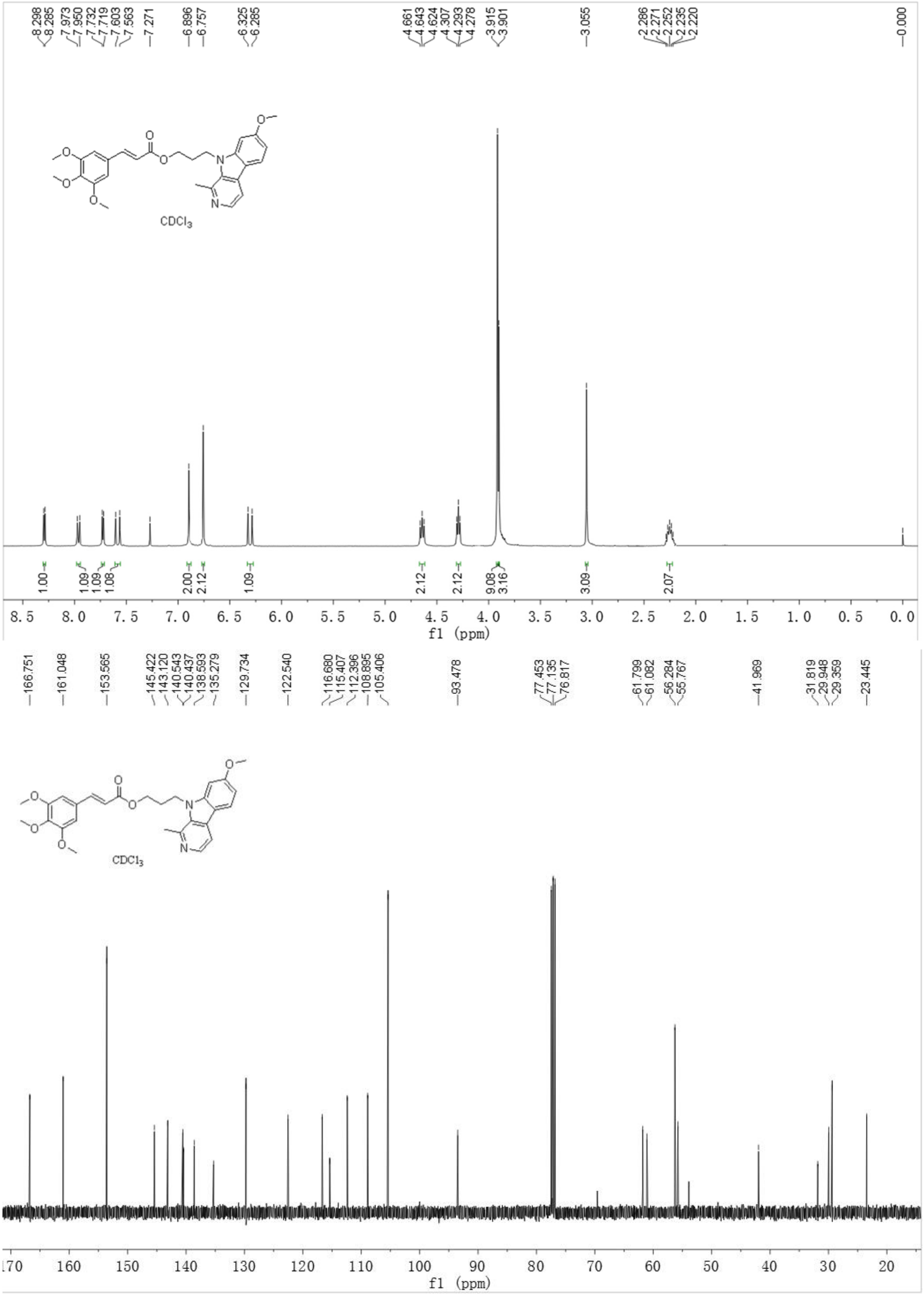

Figure S2. The ^{1}H-NMR and ^{13}C-NMR spectrum of compound **3b**.

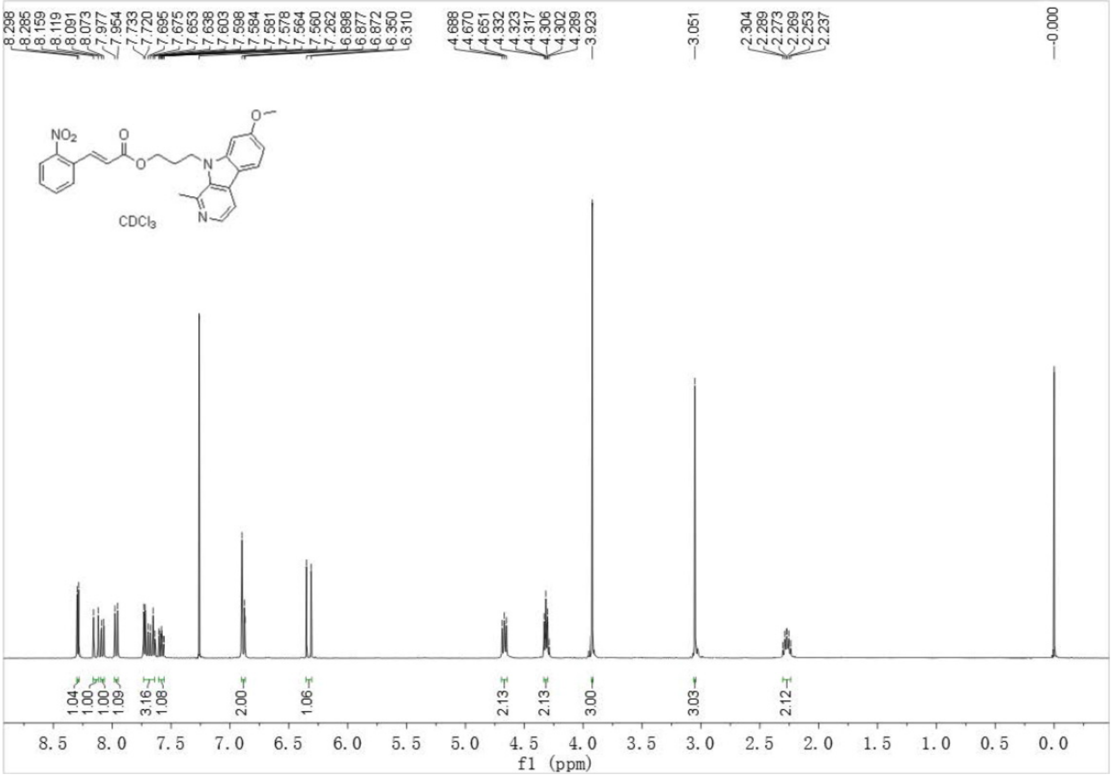

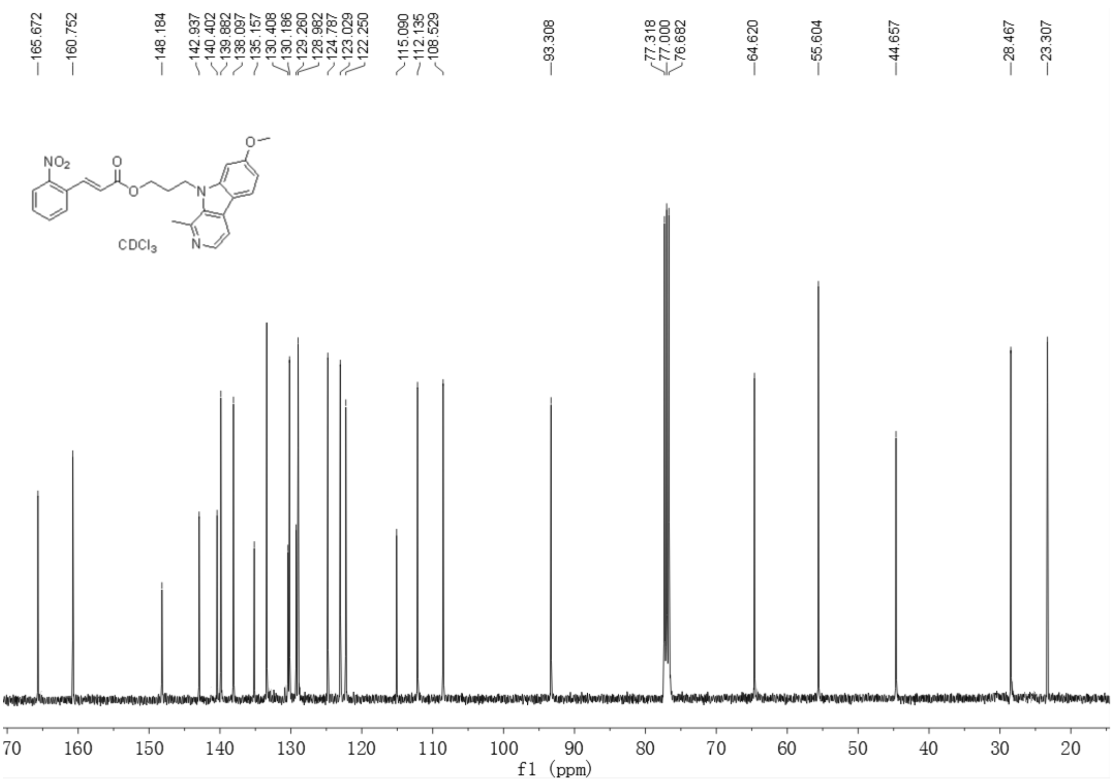

Figure S3. The ^{1}H-NMR and ^{13}C-NMR spectrum of compound **3c**.

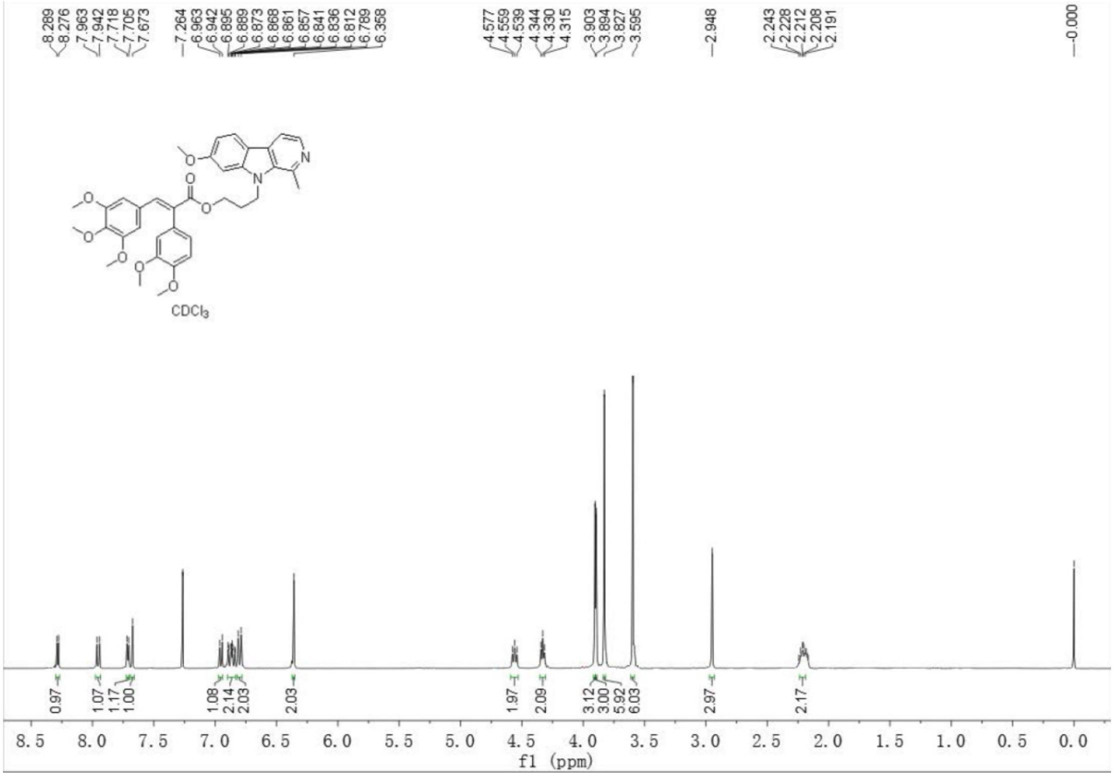

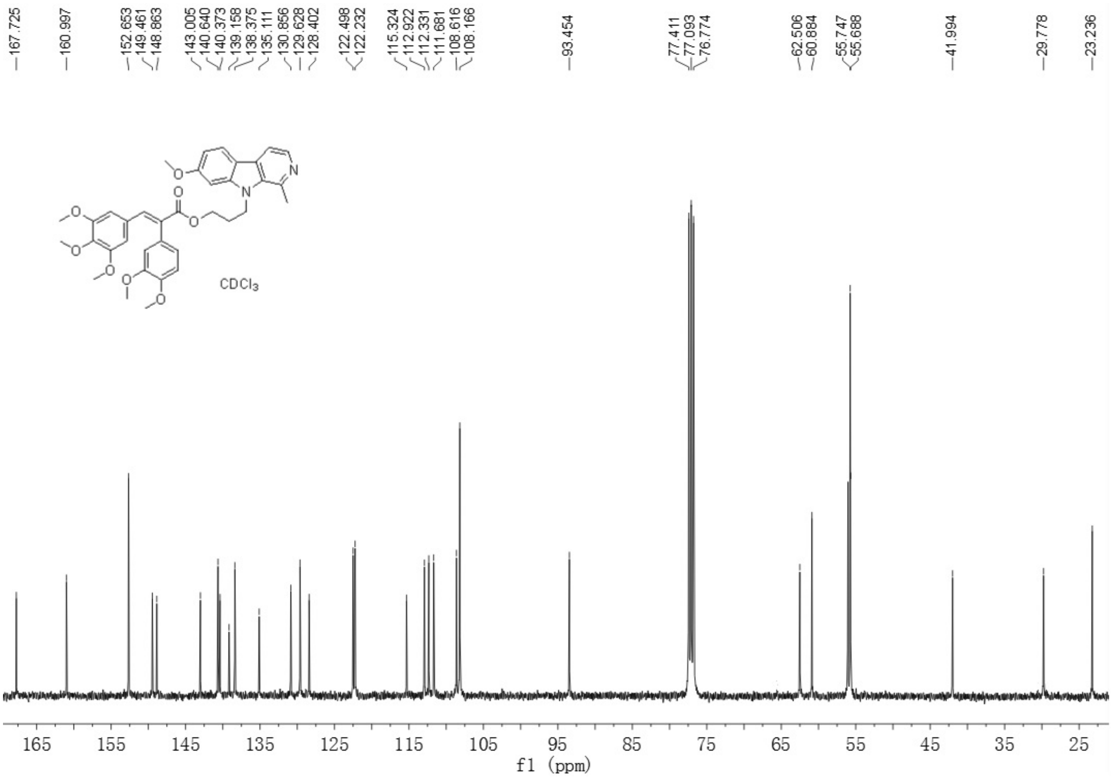

Figure S4. The ^{1}H-NMR and ^{13}C-NMR spectrum of compound **3d**.

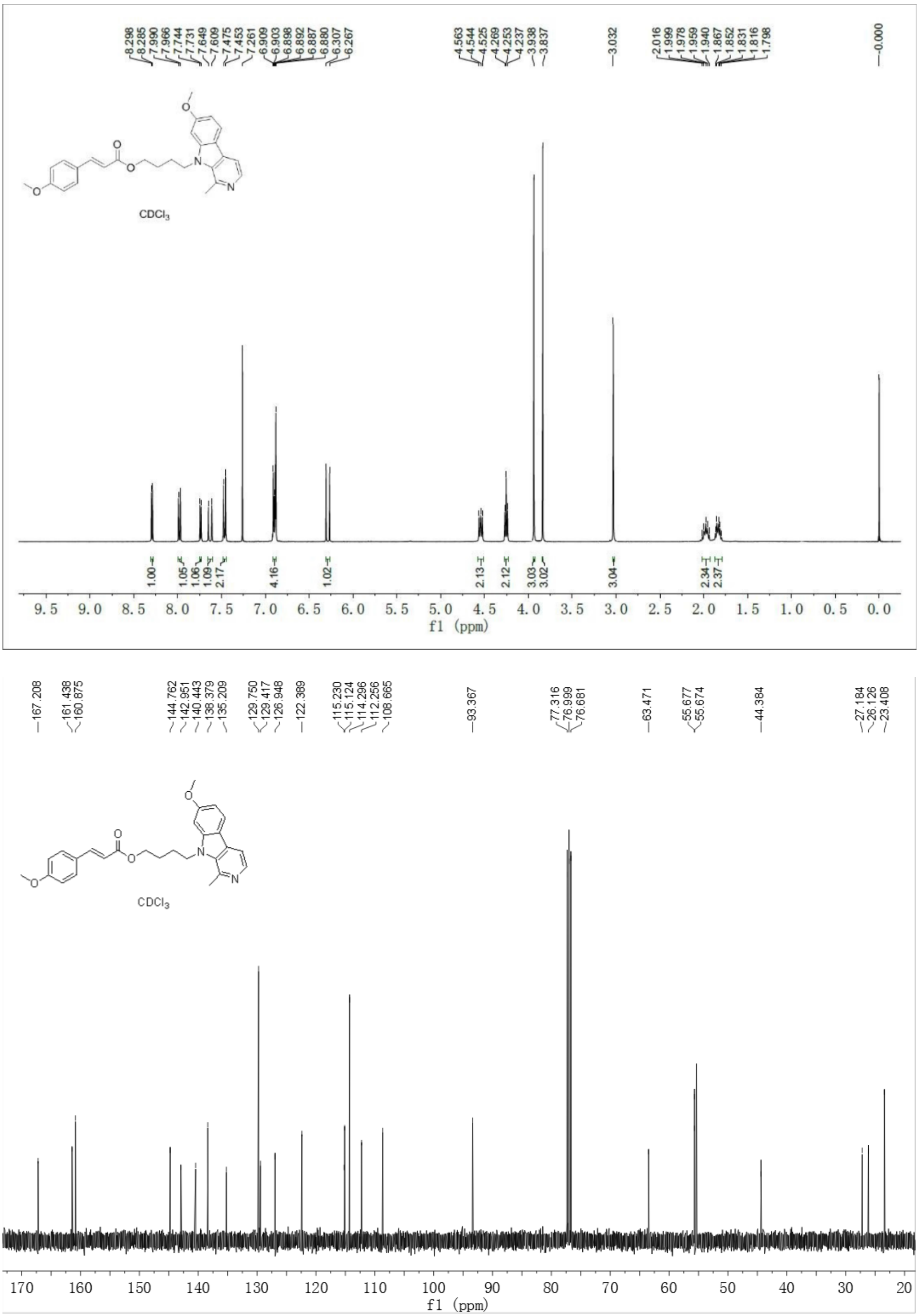

Figure S5. The ^{1}H-NMR and ^{13}C-NMR spectrum of compound **4a**.

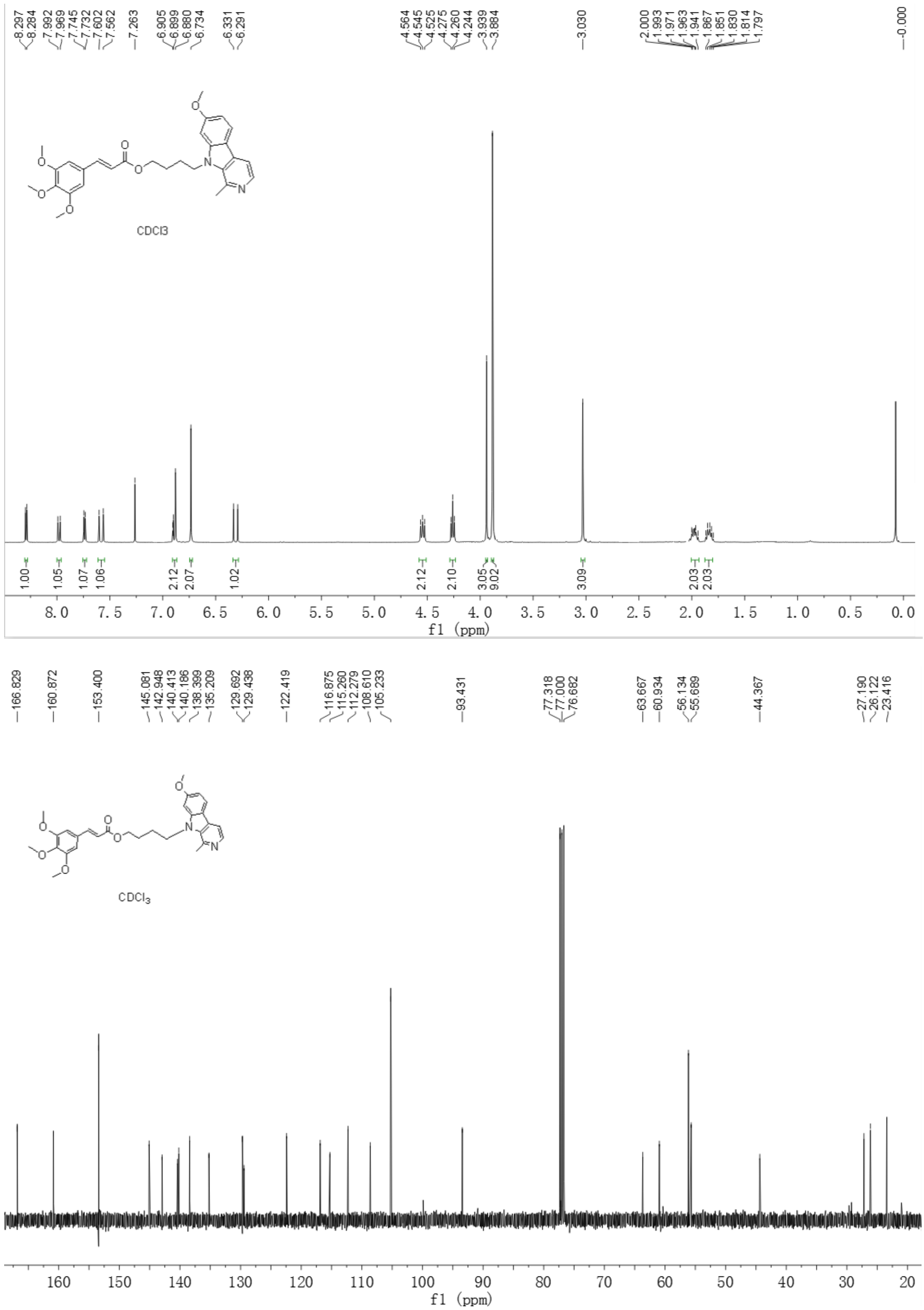

Figure S6. The ^{1}H-NMR and ^{13}C-NMR spectrum of compound **4b**.

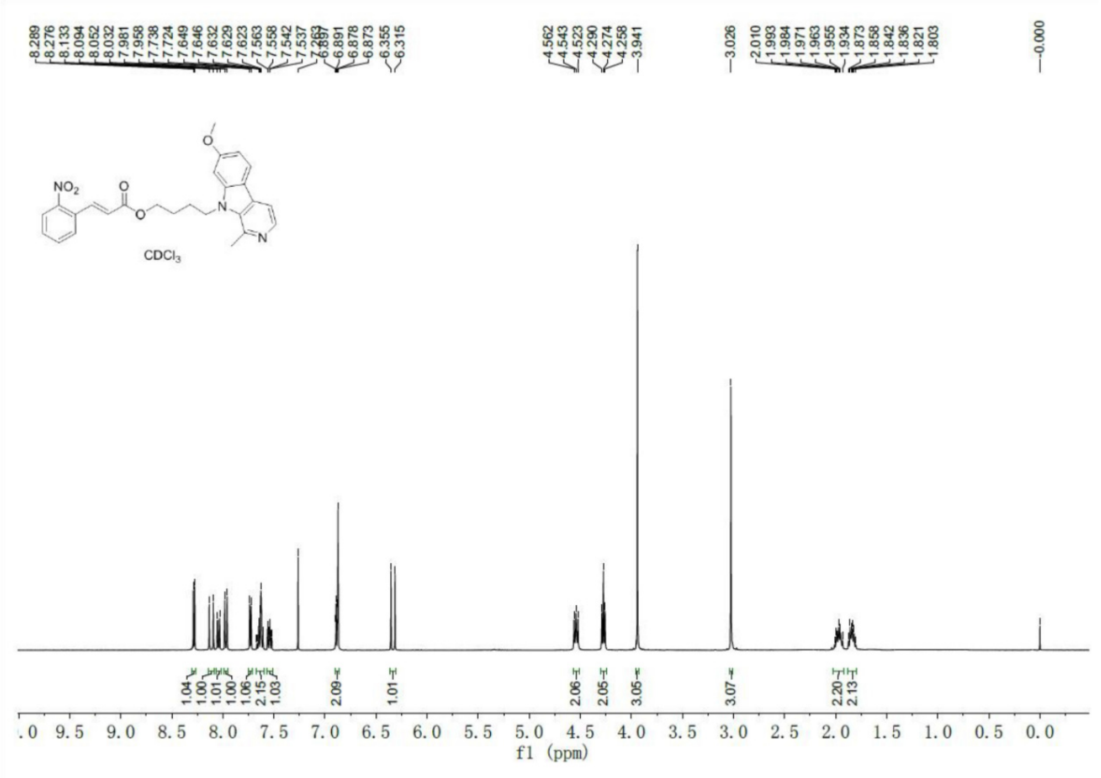

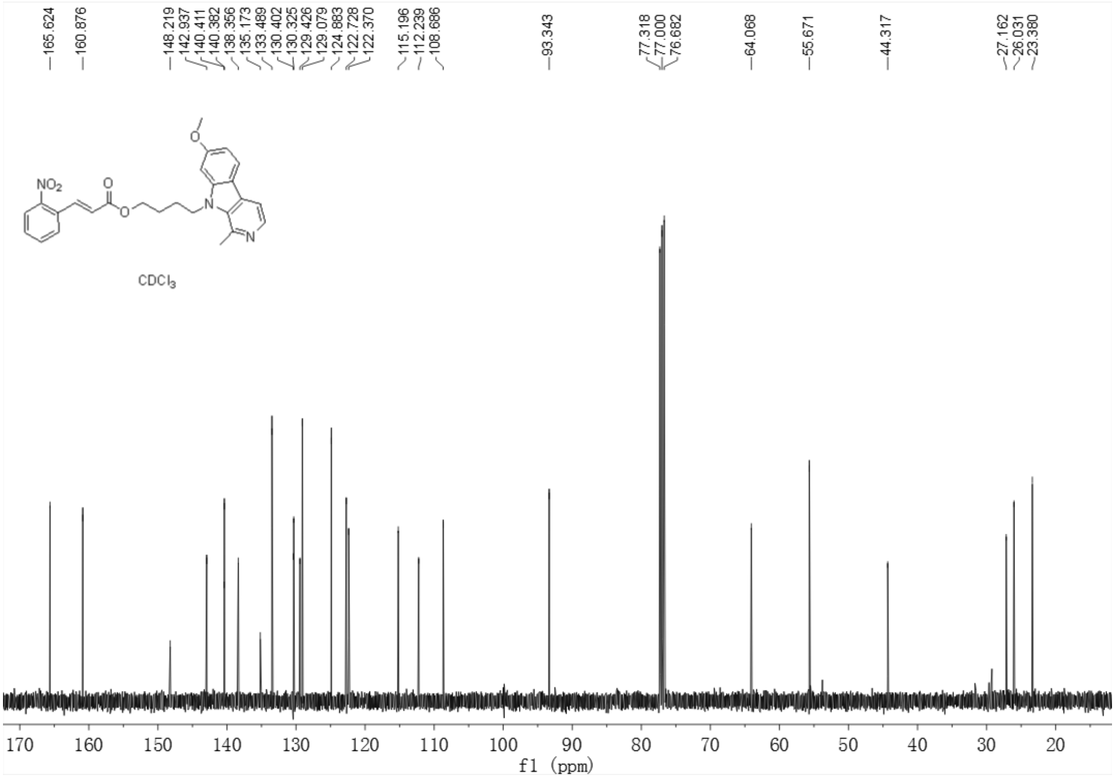

Figure S7. The ^{1}H-NMR and ^{13}C-NMR spectrum of compound **4c**.

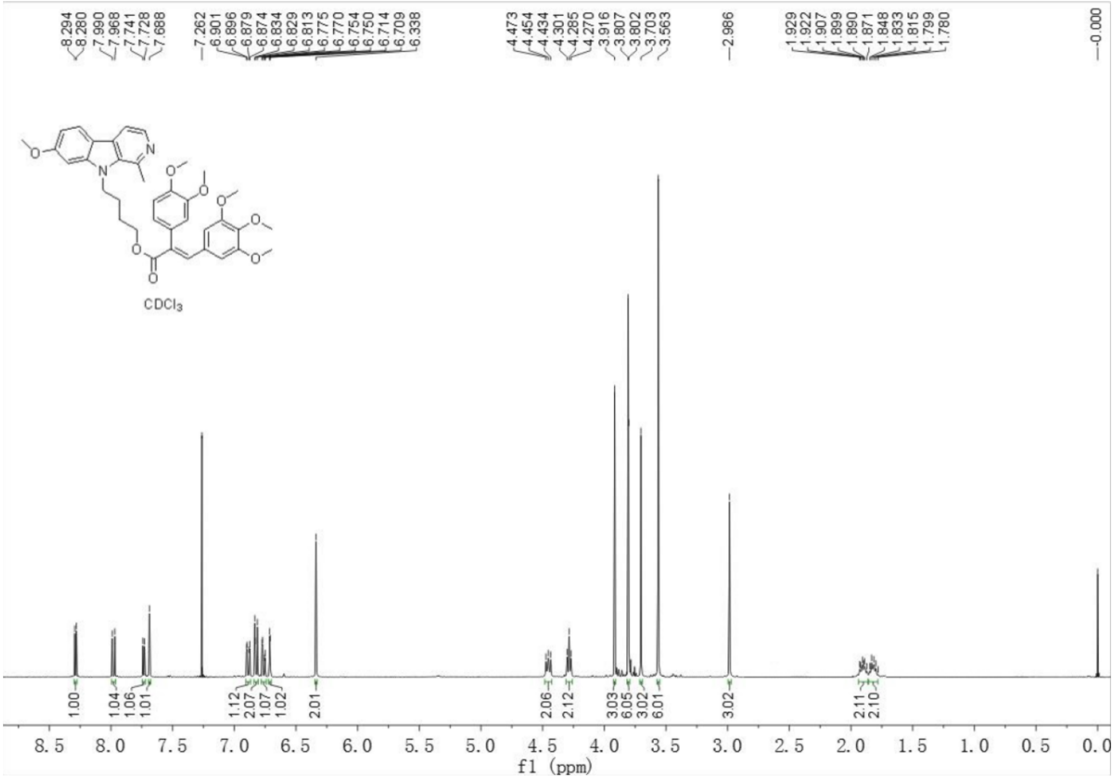

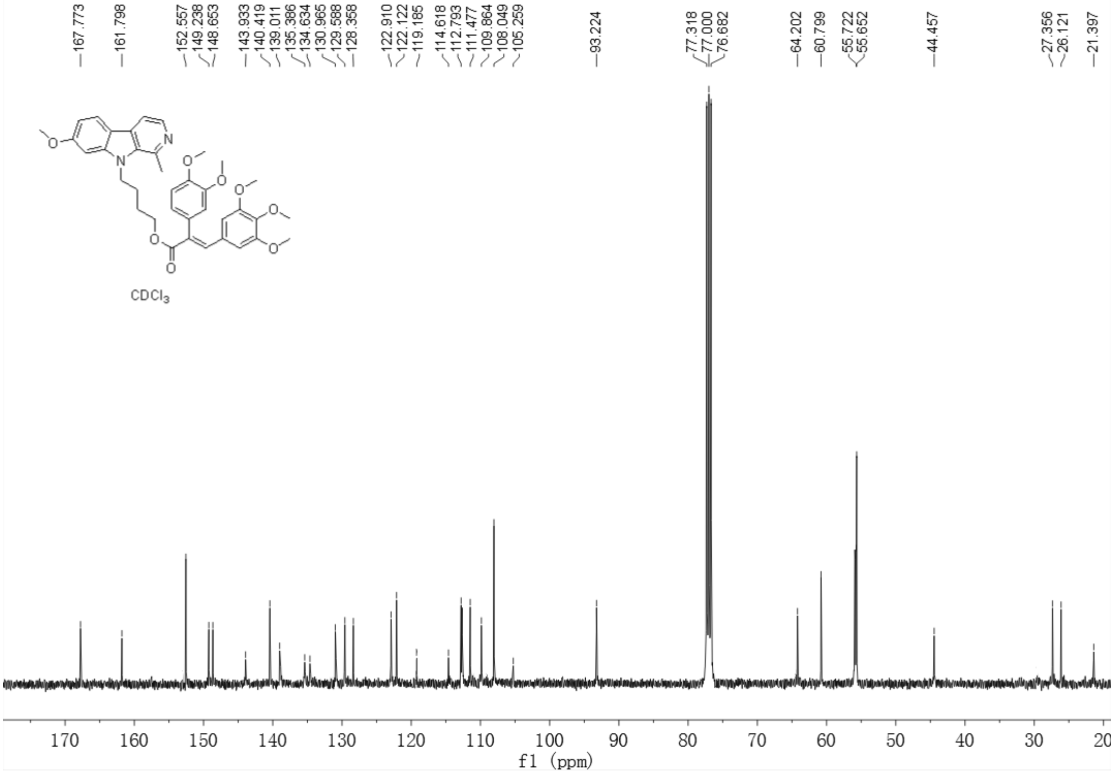

Figure S8. The ^{1}H-NMR and ^{13}C-NMR spectrum of compound **4d**.

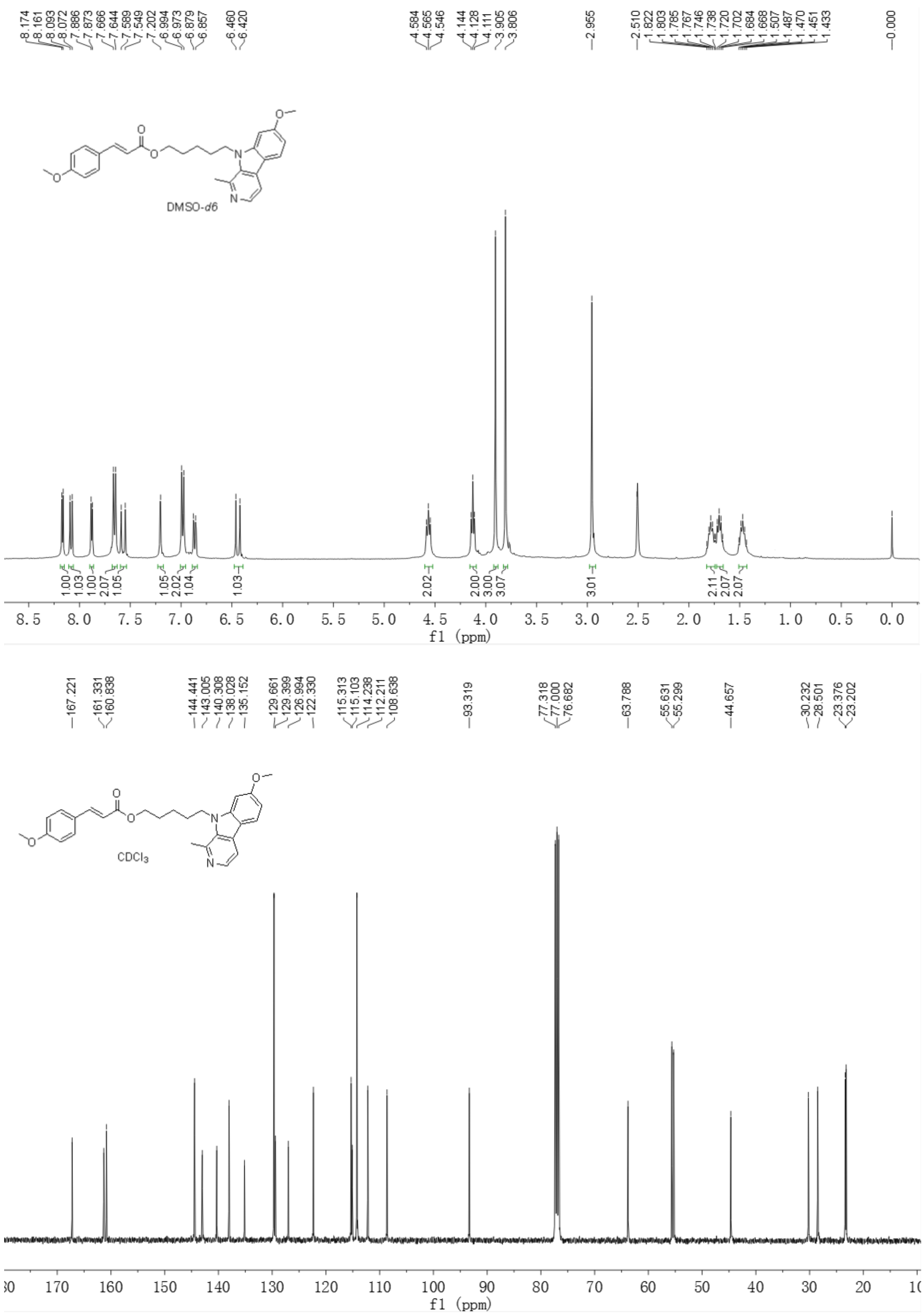

Figure S9. The ^{1}H-NMR and ^{13}C-NMR spectrum of compound **5a**.

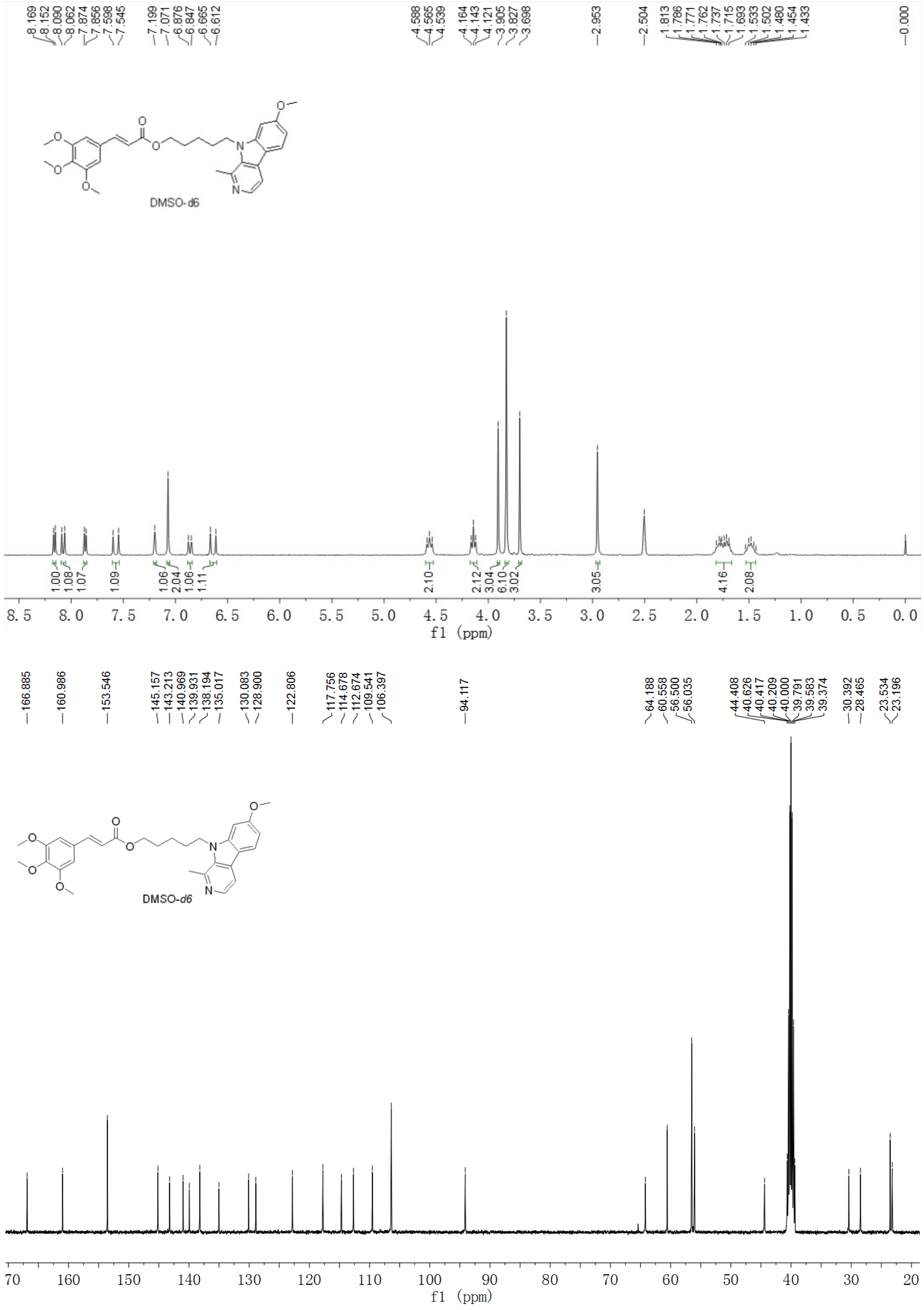

Figure S10. The ^{1}H-NMR and ^{13}C-NMR spectrum of compound **5b**.

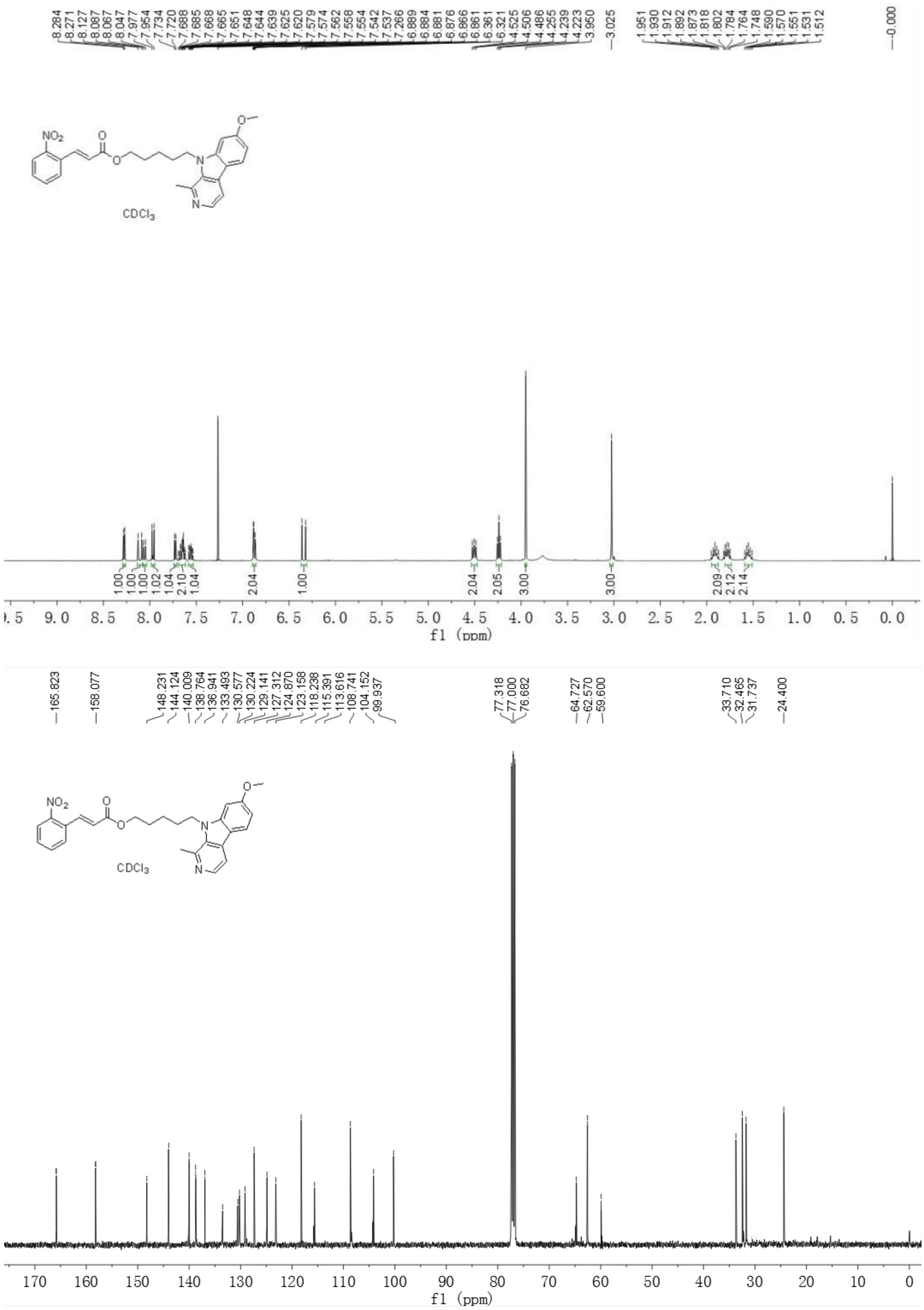

Figure S11. The ^{1}H-NMR and ^{13}C-NMR spectrum of compound **5c**.

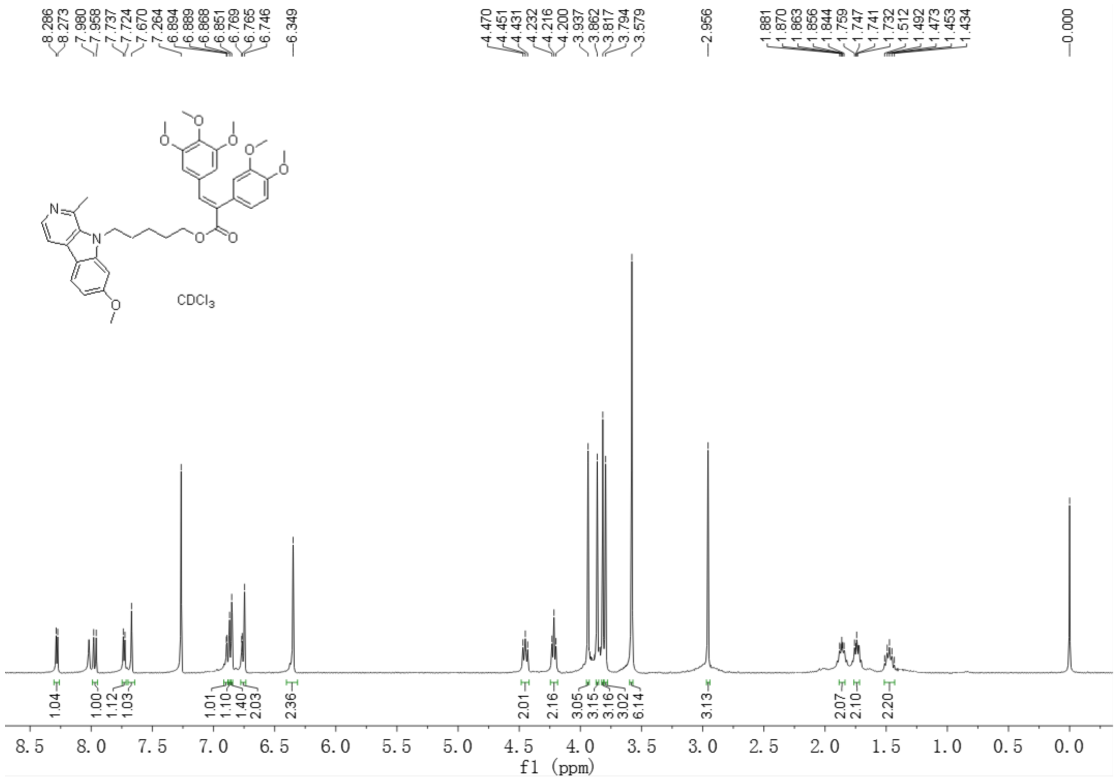

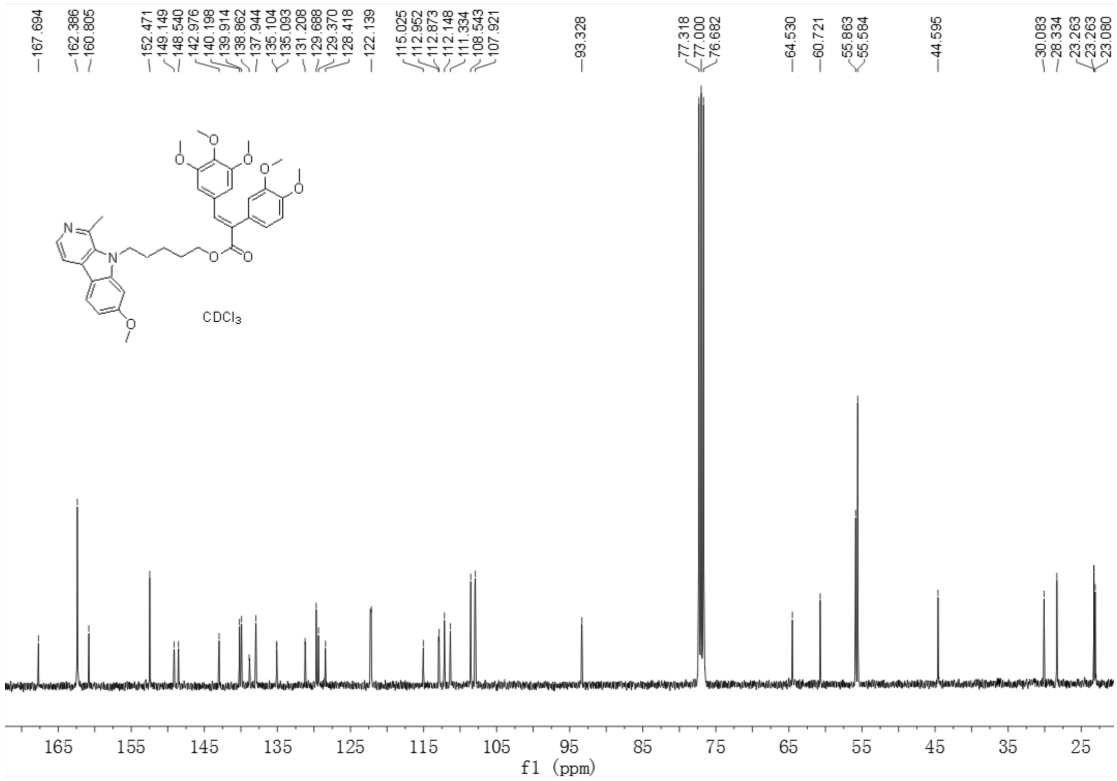

Figure S12. The ^{1}H-NMR and ^{13}C-NMR spectrum of compound **5d**.

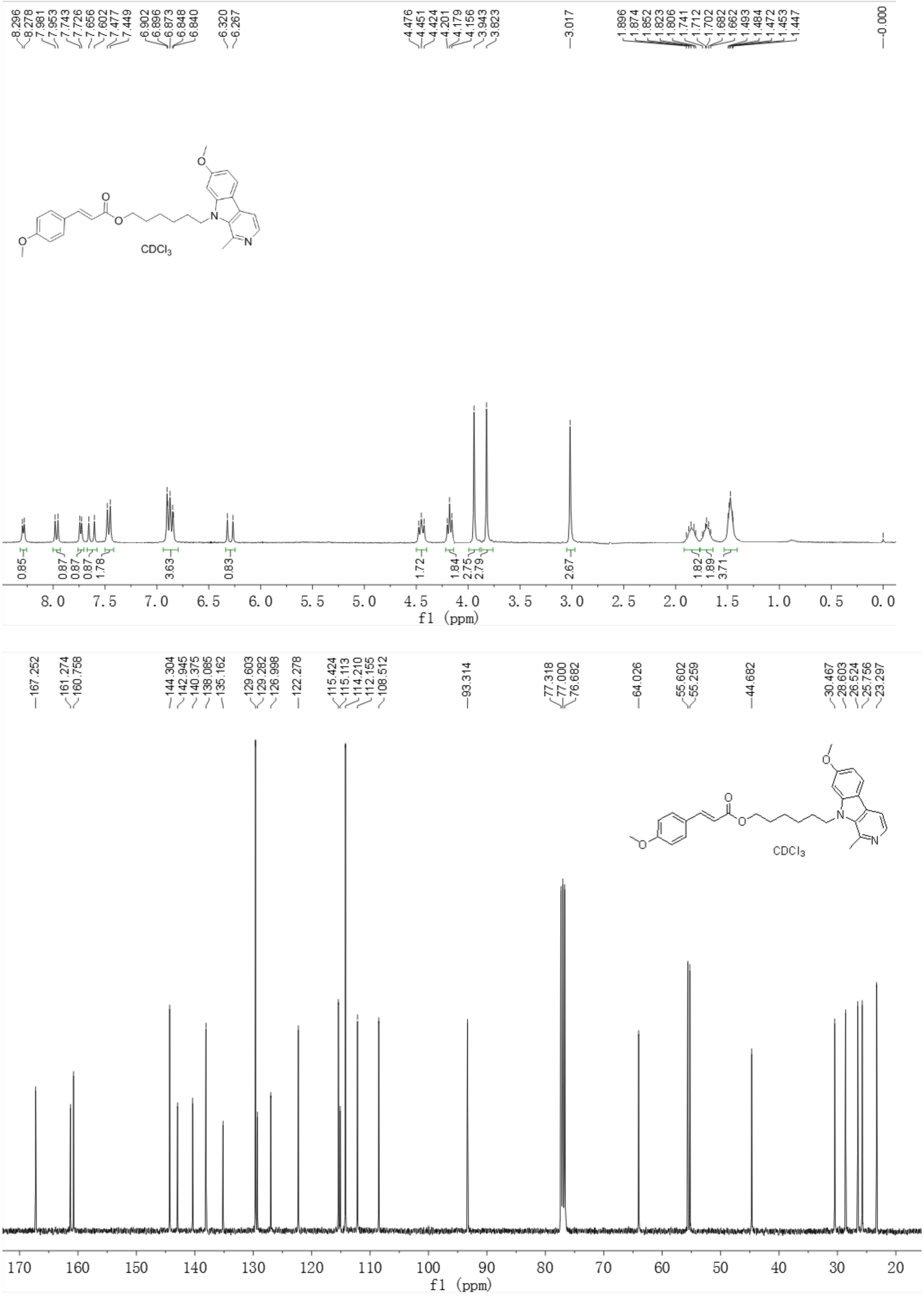

Figure S13. The ^{1}H-NMR and ^{13}C-NMR spectrum of compound **6a**.

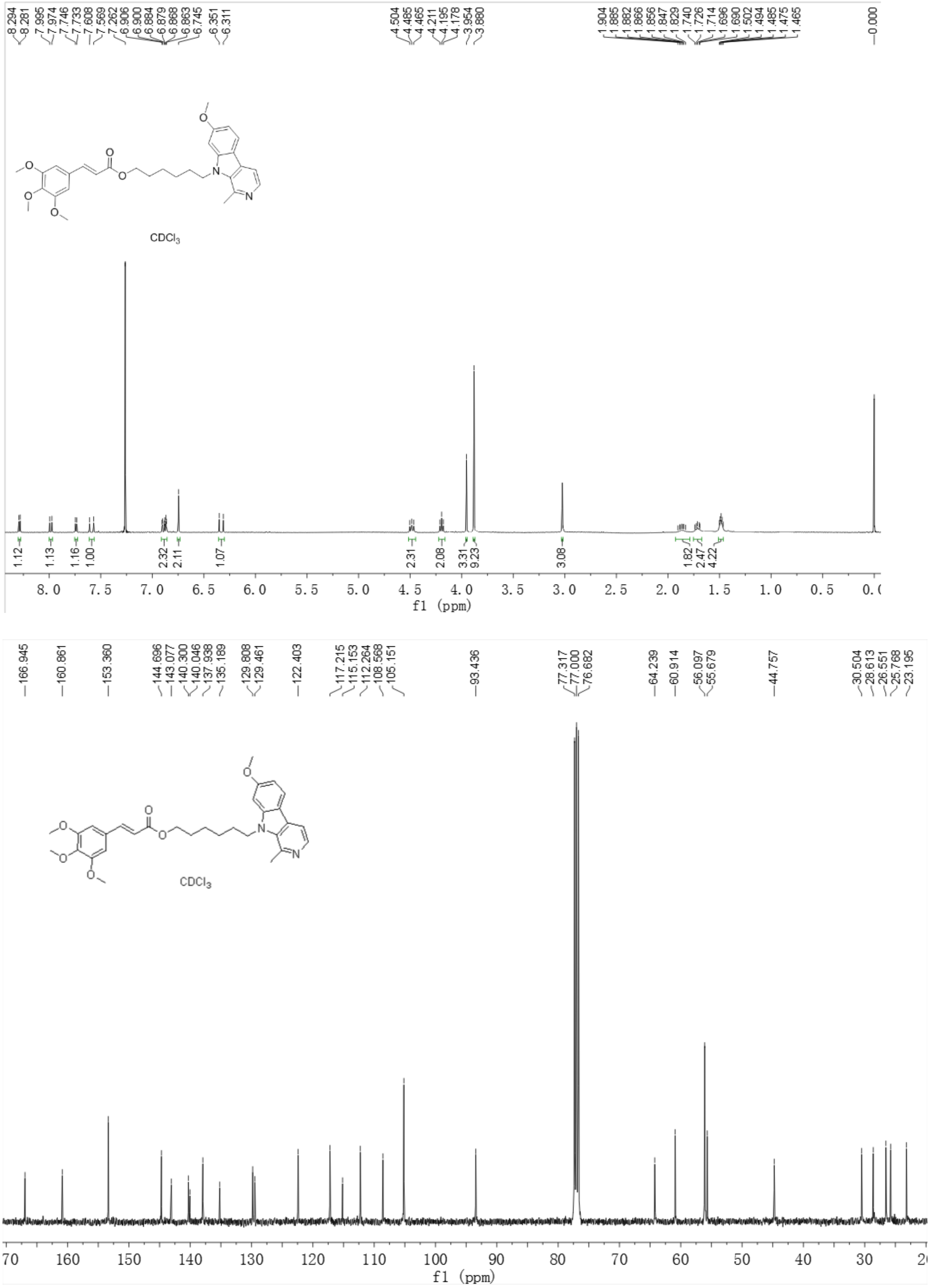

Figure S14. The ^{1}H-NMR and ^{13}C-NMR spectrum of compound **6b**.

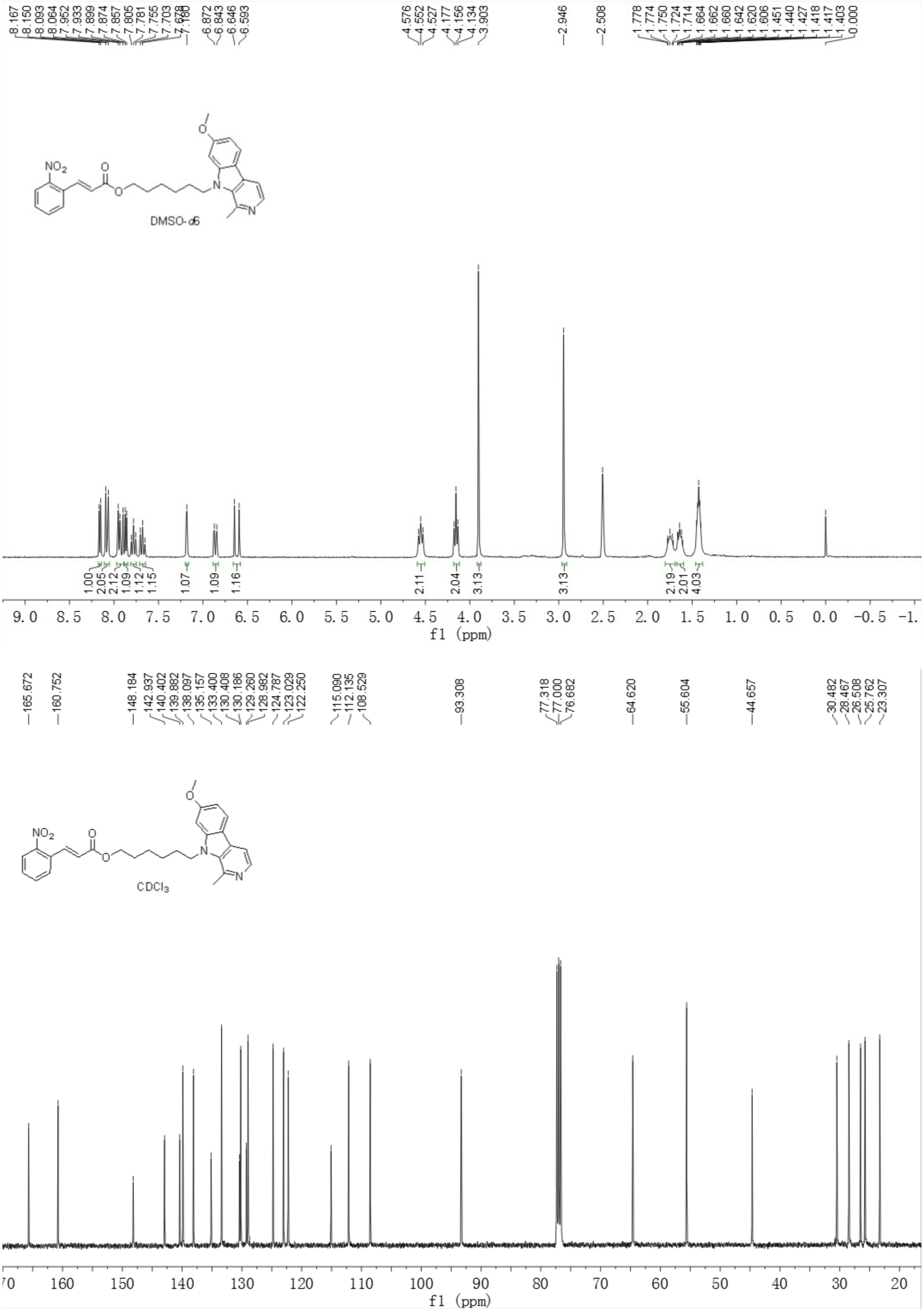

Figure S15. The ^{1}H-NMR and ^{13}C-NMR spectrum of compound **6c**.

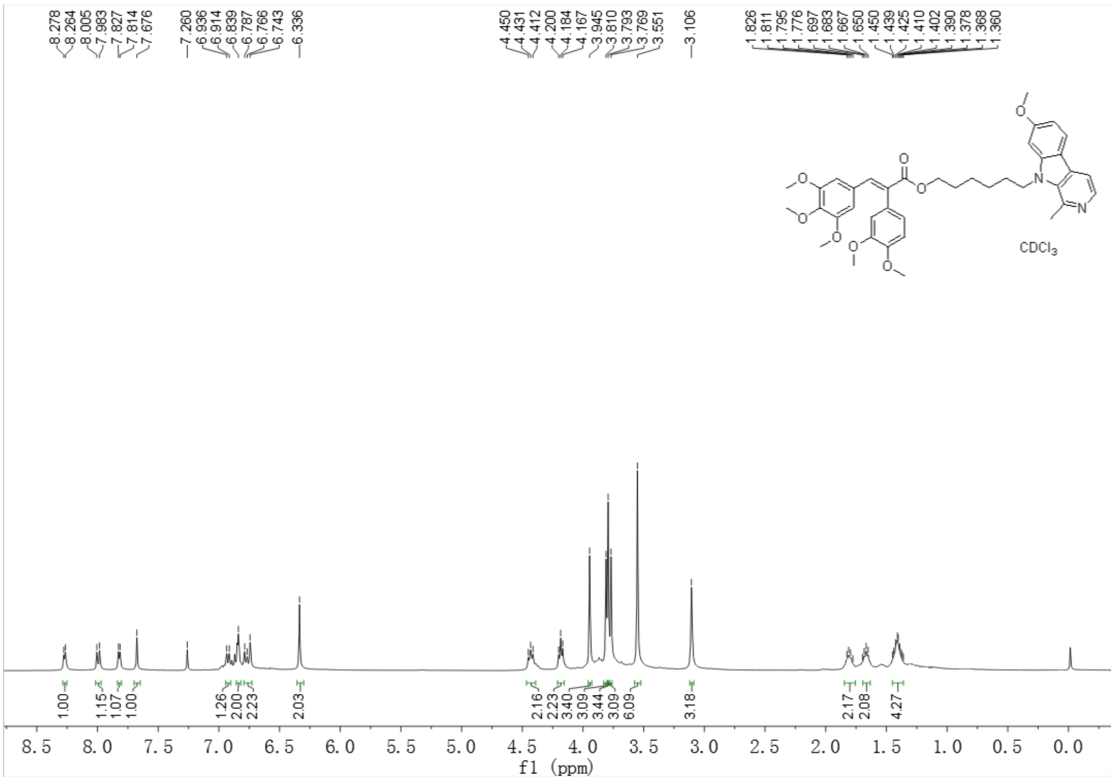

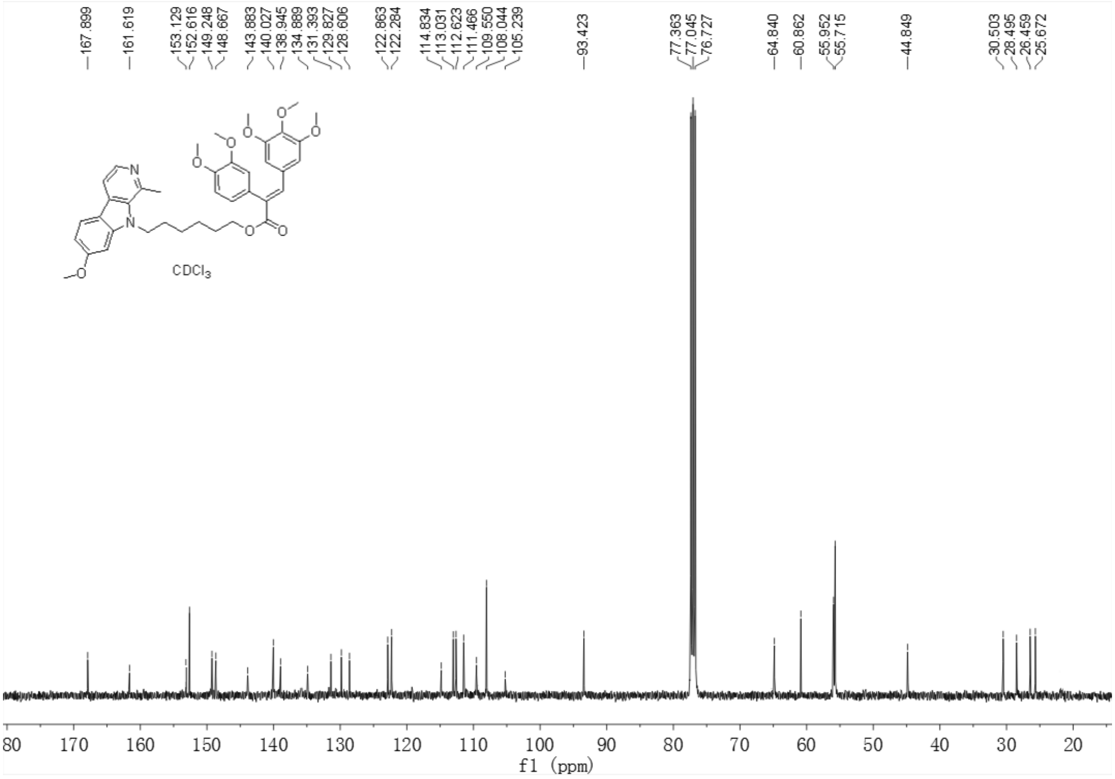

Figure S16. The ^{1}H-NMR and ^{13}C-NMR spectrum of compound **6d**.

**Table S1.** Crystal data of compound **4a**	
Empirical formula	$C_{27}H_{28}N_2O_4$
Formula weight	445.18
Temperature/K	291.7(2)
Crystal system	orthorhombic
Space group	$Pbc2_1$
a/Å	14.0624(3)
b/Å	9.3627(2)
c/Å	34.6627(7)
α/°	90
β/°	90
γ/°	90
Volume/Å^3	4563.73(16)
Z	4
ρ_{calc}g/cm^3	1.295
μ/mm^{-1}	0.703
F(000)	1892
Crystal size/mm^3	0.19 × 0.15 × 0.12
Radiation	CuKα (λ = 1.54184)
2θ range for data collection/°	8.096 to 133.18
Index ranges	−16 ≤ h ≤ 12, −11 ≤ k ≤ 8, −37 ≤ l ≤ 41
Reflections collected	32463
Independent reflections	7469 [R_{int} = 0.0375, R_{sigma} = 0.0346]
Data/restraints/parameters	7469/1/601
Goodness-of-fit on F^2	1.078
Final R indexes [I>=2σ (I)]	R_1 = 0.0771, wR_2 = 0.2129
Final R indexes [all data]	R_1 = 0.1022, wR_2 = 0.2568
Largest diff. peak/hole / e Å^{-3}	0.44/−0.25
Flack parameter	0.32(9)

Table S2. Bond length of compound **4a (Å).**

Bond	Length/Å	Bond	Length/Å
O2-C22	1.337(9)	C19-C18	1.398(10)
O2-C23	1.452(9)	C19-C14	1.384(12)
C15-O4	1.363(10)	C7-N1	1.393(11)
C15-C14	1.362(11)	C7-C9	1.372(13)
C15-C16	1.390(10)	C7-C8	1.455(12)
C22-O3	1.198(10)	C26-N1	1.500(11)
C22-C21	1.469(11)	C11-C8	1.354(14)
C17-C18	1.386(11)	C11-C10	1.352(15)
C17-C16	1.358(11)	N1-C4	1.340(12)
C20-C21	1.317(10)	C9-N2	1.287(12)
C20-C18	1.466(11)	C9-C13	1.549(13)
C23-C22	1.499(10)	C3-C4	1.344(14)
C5-C8	1.392(14)	C3-C2	1.336(14)
C5-C4	1.452(13)	O1-C2	1.332(12)
C5-C6	1.438(14)	O1-C12	1.433(15)
C25-C24	1.490(11)	C2-C1	1.538(16)
C25-C26	1.512(11)	C10-N2	1.444(14)
C1-C6	1.316(15)		

Table S3. Bond angle of compound **4a** (°).

Bond	Angle/°	Bond	Angle/°
C22-O2-C23	115.7(6)	C3-C2-C1	118.6(10)
O4-C15-C16	115.8(7)	O1-C2-C3	131.9(13)
C14-C15-O4	125.1(7)	O1-C2-C1	109.5(11)
C14-C15-C16	119.1(8)	C11-C10-N2	126.0(10)
O2-C22-C21	113.3(6)	C9-N2-C10	112.1(10)
O3-C22-O2	122.7(7)	C6-C1-C2	118.3(10)
O3-C22-C21	124.0(7)	C1-C6-C5	121.2(10)
C16-C17-C18	120.8(7)	O2-C22-C21-C20	−174.3(7)
C21-C20-C18	129.3(7)	O2-C23-C24-C25	−179.3(7)
O2-C23-C24	110.3(6)	O4-C15-C14-C19	−179.1(7)
C8-C5-C4	105.4(8)	O4-C15-C16-C17	179.8(7)
C8-C5-C6	135.4(9)	C22-O2-C23-C24	174.8(7)
C6-C5-C4	119.2(10)	C23-O2-C22-O3	−3.1(11)
C20-C21-C22	120.2(7)	C23-O2-C22-C21	177.4(6)
C24-C25-C26	115.0(7)	O3-C22-C21-C20	6.2(13)
C14-C19-C18	121.8(8)	C21-C20-C18-C17	1.8(13)
C25-C24-C23	109.7(6)	C21-C20-C18-C19	−177.9(8)
C17-C18-C20	124.6(7)	C25-C26-N1-C7	83.5(10)
C17-C18-C19	117.2(7)	C25-C26-N1-C4	−75.9(10)
C19-C18-C20	118.2(7)	C24-C25-C26-N1	−176.7(8)
C15-C14-C19	119.6(7)	C18-C17-C16-C15	−0.5(12)
N1-C7-C8	104.0(9)	C18-C20-C21-C22	−179.2(7)
C9-C7-N1	137.8(9)	C18-C19-C14-C15	−0.4(13)
C9-C7-C8	118.1(8)	C14-C15-C16-C17	1.4(12)
N1-C26-C25	111.9(6)	C14-C19-C18-C17	1.3(12)
C10-C11-C8	118.9(9)	C14-C19-C18-C20	−179.0(7)
C7-N1-C26	126.4(8)	C7-N1-C4-C5	0.1(8)
C4-N1-C7	112.4(8)	C7-N1-C4-C3	178.7(9)
C4-N1-C26	118.3(8)	C7-C9-N2-C10	−0.3(12)
C7-C9-C13	122.8(10)	C26-C25-C24-C23	179.8(8)
N2-C9-C7	127.6(10)	C26-N1-C4-C5	162.2(6)
N2-C9-C13	109.5(10)	C26-N1-C4-C3	−19.2(13)
C17-C16-C15	121.5(8)	C11-C10-N2-C9	−0.8(13)
C5-C8-C7	109.8(8)	N1-C7-C9-N2	177.4(8)
C11-C8-C5	132.7(9)	N1-C7-C9-C13	−0.2(14)
C11-C8-C7	117.4(10)	N1-C7-C8-C5	−1.4(8)
C2-C3-C4	124.0(12)	N1-C7-C8-C11	−177.5(7)
C2-O1-C12	111.6(10)	C9-C7-N1-C26	23.2(14)
N1-C4-C5	108.3(9)	C9-C7-N1-C4	−176.4(9)
N1-C4-C3	132.9(11)	C9-C7-C8-C5	176.5(7)
C3-C4-C5	118.7(10)	C9-C7-C8-C11	0.4(10)
C16-C15-C14-C19	−0.9(12)	C4-C5-C6-C1	−0.5(13)
C16-C17-C18-C20	179.5(7)	C4-C3-C2-O1	179.7(9)
C16-C17-C18-C19	−0.8(11)	C4-C3-C2-C1	−0.1(13)
C8-C5-C4-N1	−1.0(8)	C2-C3-C4-C5	−0.9(13)
C8-C5-C4-C3	−179.8(7)	C2-C3-C4-N1	−179.4(9)
C8-C5-C6-C1	−179.2(9)	C2-C1-C6-C5	−0.4(14)
C8-C7-N1-C26	−159.6(7)	C10-C11-C8-C5	−176.3(9)
C8-C7-N1-C4	0.8(8)	C10-C11-C8-C7	−1.3(12)
C8-C7-C9-N2	0.5(12)	C13-C9-N2-C10	177.6(7)
C8-C7-C9-C13	−177.1(7)	C6-C5-C8-C7	−179.8(9)
C8-C11-C10-N2	1.6(14)	C6-C5-C8-C11	−4.5(16)
C3-C2-C1-C6	0.7(13)	C6-C5-C4-N1	−180.0(7)
O1-C2-C1-C6	−179.1(9)	C6-C5-C4-C3	1.2(11)

C4-C5-C8-C7	1.5(8)	C12-O1-C2-C3	−7.7(15)
C4-C5-C8-C11	176.7(8)	C12-O1-C2-C1	172.0(8)
